# Adverse pregnancy and perinatal outcomes in women with polycystic ovary syndrome undergoing assisted reproductive technology: a systematic review and meta-analysis

**DOI:** 10.3389/fmed.2025.1656389

**Published:** 2025-10-10

**Authors:** Nian Xie, Wenwen Zhao

**Affiliations:** Teaching and Research Section of Clinical Nursing, The Second Xiangya Hospital, Central South University, Changsha, China

**Keywords:** polycystic ovary syndrome, pregnancy outcomes, assisted reproductive technology, miscarriage, preterm birth, meta-analysis

## Abstract

**Background:**

The growing application of assisted reproductive technology (ART) has enabled more women with polycystic ovary syndrome (PCOS) to achieve pregnancy. However, the causal association between PCOS and reproductive outcomes remains uncertain. This study conducted a meta-analysis of cohort studies to explore the association between PCOS and adverse pregnancy and perinatal outcomes.

**Methods:**

A comprehensive search was carried out in PubMed, Web of Science, Embase, and the Cochrane Library to identify studies published prior to March 22, 2025. Cohort studies evaluating differences in adverse pregnancy and perinatal outcomes between women with PCOS and those without PCOS undergoing ART were included. Meta-analysis was conducted using R 4.3.2 and STATA 12.0 to estimate risk ratios (RRs) and 95% confidence intervals (CIs) for the association between PCOS and adverse outcomes. Study heterogeneity was assessed through Cochran’s Q test, *I*^2^ statistics, and 95% prediction intervals (PIs). Additionally, subgroup analysis, sensitivity analysis, and publication bias evaluation were performed to ensure the reliability and validity of the results.

**Results:**

This meta-analysis included 18 cohort studies, comprising 16,365 women with PCOS and 111,503 controls. Women with PCOS undergoing ART were found to have significantly higher clinical pregnancy rate (RR = 1.158, 95% CI: 1.004–1.335; 95% PI: 0.751–1.785) and live birth rate (RR = 1.084, 95% CI: 1.027–1.144; 95% PI: 0.827–1.361) compared to those without PCOS. However, these patients also exhibited an increased risk of miscarriage (RR = 1.301, 95% CI: 1.181–1.433; 95% PI: 0.917–1.957), gestational diabetes mellitus (GDM), hypertensive disorders of pregnancy (HDP), gestational hypertension, preterm premature rupture of membranes (PPROM), preterm birth (PTB) (RR = 1.259, 95% CI: 1.152–1.376; 95% PI: 1.143–1.387), and very preterm birth (VPTB), while showing a reduced risk of cesarean delivery (RR = 0.898, 95% CI: 0.810–0.994; 95% PI: 0.717–1.124). No significant differences were identified between PCOS and control groups regarding the risks of low birth weight, very low birth weight, macrosomia, small for gestational age, very small for gestational age, large for gestational age, or fetal malformation (all *p* > 0.05). Subgroup analysis of patients undergoing frozen embryo transfer (FET) yielded consistent results.

**Conclusion:**

PCOS may affect pregnancy and perinatal outcomes in women undergoing ART, with an increased risk of miscarriage, GDM, HDP, gestational hypertension, PPROM, PTB, and VPTB. These results underscore the importance of tailored reproductive strategies and specialized perinatal management for women affected by PCOS.

## 1 Introduction

Polycystic ovary syndrome (PCOS), a prevalent endocrine disorder, affects an estimated 5–20% of women of reproductive age globally, making it one of the leading causes of female infertility (1). This complex disorder is marked by irregular ovarian function, an imbalance in androgen levels, and the presence of cyst-like structures in the ovaries (2). Beyond its reproductive implications, this syndrome is associated with metabolic disturbances and psychological comorbidities, exerting a multifaceted impact across the lifespan (3). Infertility in women with PCOS is frequently attributed to anovulation (4), often necessitating the use of assisted reproductive technologies (ART) to achieve pregnancy. Techniques such as *in vitro* fertilization (IVF) and intracytoplasmic sperm injection (ICSI) have demonstrated efficacy in improving fertility outcomes for affected individuals (4, 5). However, the underlying pathophysiological complexities of PCOS may predispose patients to heightened risks during pregnancy and childbirth, with potential adverse effects on maternal health and neonatal outcomes.

Existing evidence indicates that women with PCOS are at an elevated risk of adverse pregnancy and perinatal outcomes, including preterm birth (PTB), miscarriage, gestational hypertension, and gestational diabetes mellitus (GDM) (6–8), irrespective of whether conception occurs naturally or through ART. Recent investigations further suggested that frozen embryo transfer (FET) cycles in PCOS patients were associated with an increased likelihood of neonatal complications, such as PTB (9). A multicenter randomized controlled trial involving 1,508 infertile women with PCOS after their first IVF cycle demonstrated that FET was associated with a significantly higher live birth rate compared with fresh embryo transfer (10). However, the heterogeneous nature of PCOS, coupled with its metabolic and hormonal complexities, renders its impact on pregnancy outcomes following IVF contentious. For instance, Sterling et al. (11) identified an increased risk of PTB and large for gestational age (LGA) among women with PCOS undergoing fresh embryo transfer, while no significant differences were observed for preterm premature rupture of membranes (PPROM). However, Qiu et al. (12) reported no differences in neonatal birth weight but noted a higher incidence of very preterm birth (VPTB) and PPROM in PCOS patients undergoing FET. These conflicting findings underscore the need for a meta-analysis to clarify the association between PCOS and adverse maternal and neonatal outcomes in ART-conceived pregnancies.

While previous meta-analyses have explored the association between PCOS and adverse pregnancy or perinatal outcomes (13–15), the inclusion of case–control and cross-sectional studies has limited the ability to establish a clear causal relationship. Therefore, we performed a meta-analysis focused exclusively on cohort studies to evaluate the risks of maternal and neonatal complications in women with PCOS undergoing ART, with a specific emphasis on comparing outcomes between FET and fresh embryo transfer cycles. Importantly, data for this analysis were extracted directly from logistic regression models reported in the included studies, enhancing the reliability and precision of the findings. This study aimed to provide robust evidence to better elucidate the causal relationship between PCOS and adverse reproductive outcomes following ART.

## 2 Materials and methods

### 2.1 Study design

This systematic review and meta-analysis was conducted in accordance with the Preferred Reporting Item for Systematic Reviews and Meta-analysis (PRISMA) guidelines (16). The study protocol was registered in the International Prospective Register of Systematic Reviews (PROSPERO) under the identifier CRD420251079585.

### 2.2 Search strategy

To identify high-quality studies, a systematic search was conducted across 4 major electronic databases, including Web of Science, PubMed, Embase, and the Cochrane Library, from their inception to March 22, 2025. The search strategy incorporated a combination of terms, encompassing (“polycystic ovary syndrome” OR “PCOS” OR “polycystic ovarian syndrome” OR “sclerocystic ovary syndrome”) AND (“preterm birth” OR “low birth weight” OR “macrosomia” OR “small for gestational age” OR “large for gestational age” OR “gestational diabetes mellitus” OR “hypertensive disorders of pregnancy” OR “cesarean delivery” OR “preterm premature rupture of membranes” OR “malformations” OR “clinical pregnancy rate” OR “miscarriage” OR “live birth rate” OR “pregnancy outcomes” OR “obstetric outcomes” OR “reproductive outcomes” OR “fertility outcomes”) AND (“cohort study” OR “cohort studies” OR “retrospective” OR “prospective”). Detailed search methodologies tailored to each database were outlined in Supplementary File 1. No restrictions on language were applied during the search process. Additionally, reference lists of relevant original studies and review articles were manually screened to identify any additional eligible studies.

### 2.3 Inclusion and exclusion criteria

Eligible studies were identified according to the following inclusion criteria: (1) the use of a cohort design, either prospective or retrospective; (2) the exposed population consisted of women diagnosed with PCOS who underwent ART; (3) the comparison group included women without PCOS who also underwent ART; (4) the study provided risk estimates, such as risk ratios (RRs) or odds ratios (ORs), accompanied by 95% confidence intervals (CIs), evaluating the relationship between PCOS and adverse pregnancy or perinatal outcomes; (5) no restrictions were applied to the language of the study. Studies were excluded if they met any of the following criteria: (1) employed a case–control or cross-sectional design; (2) analyzed a mixed population without distinguishing outcomes from natural conception versus ART; (3) failed to provide data on relevant outcomes; (4) case reports, conference abstracts, reviews, animal studies, editorials, or commentaries.

### 2.4 Data extraction and quality assessment

Following the predefined eligibility criteria, two reviewers independently evaluated the titles, abstracts, and full texts to determine suitability for inclusion. Data extracted from the eligible studies included the following variables: name of the first author, year of publication, study location, research design, sample size, selection of the controls, maternal age and body mass index (BMI) for PCOS patients and controls, type of ART utilized, adjusted confounding factors, and outcomes included in the meta-analysis. The methodological quality of the included cohort studies was appraised using the Newcastle-Ottawa Scale (NOS) (17), which evaluates studies across three domains: selection of participants, comparability of groups, and assessment of exposure. Each domain contains specific criteria scored on a scale of one or two points, depending on the degree to which standards are met. Studies were categorized according to their NOS scores as low quality (0–3 points), moderate quality (4–6 points), or high quality (7–9 points) (18).

### 2.5 Statistical analysis

To examine the association between PCOS and adverse pregnancy or perinatal outcomes, RRs with 95% CIs were calculated. Heterogeneity among studies was assessed using Cochran’s Q test, complemented by the *I*^2^ and Tau^2^ statistics, as well as the 95% prediction interval (PI) (19, 20). When the data demonstrated homogeneity (*p* < 0.10 or *I*^2^ > 50%), a random-effects model, utilizing the DerSimonian-Laird method, was applied to estimate the association. In contrast, when no significant heterogeneity was detected, the Mantel–Haenszel fixed-effects model was used (21). To ensure the reliability of the findings, sensitivity analyses were performed by systematically excluding individual studies. Additionally, subgroup analyses were conducted for categories with at least two studies to explore the influence of ART type on pooled RR estimates. Publication bias was assessed using Begg’s (22) and Egger’s (23) tests. A two-sided *p*-value of less than 0.05 was considered statistically significant. All statistical analyses were conducted using STATA (version 12.0) and R (version 4.3.2).

## 3 Results

### 3.1 Search results

**Figure 1:**
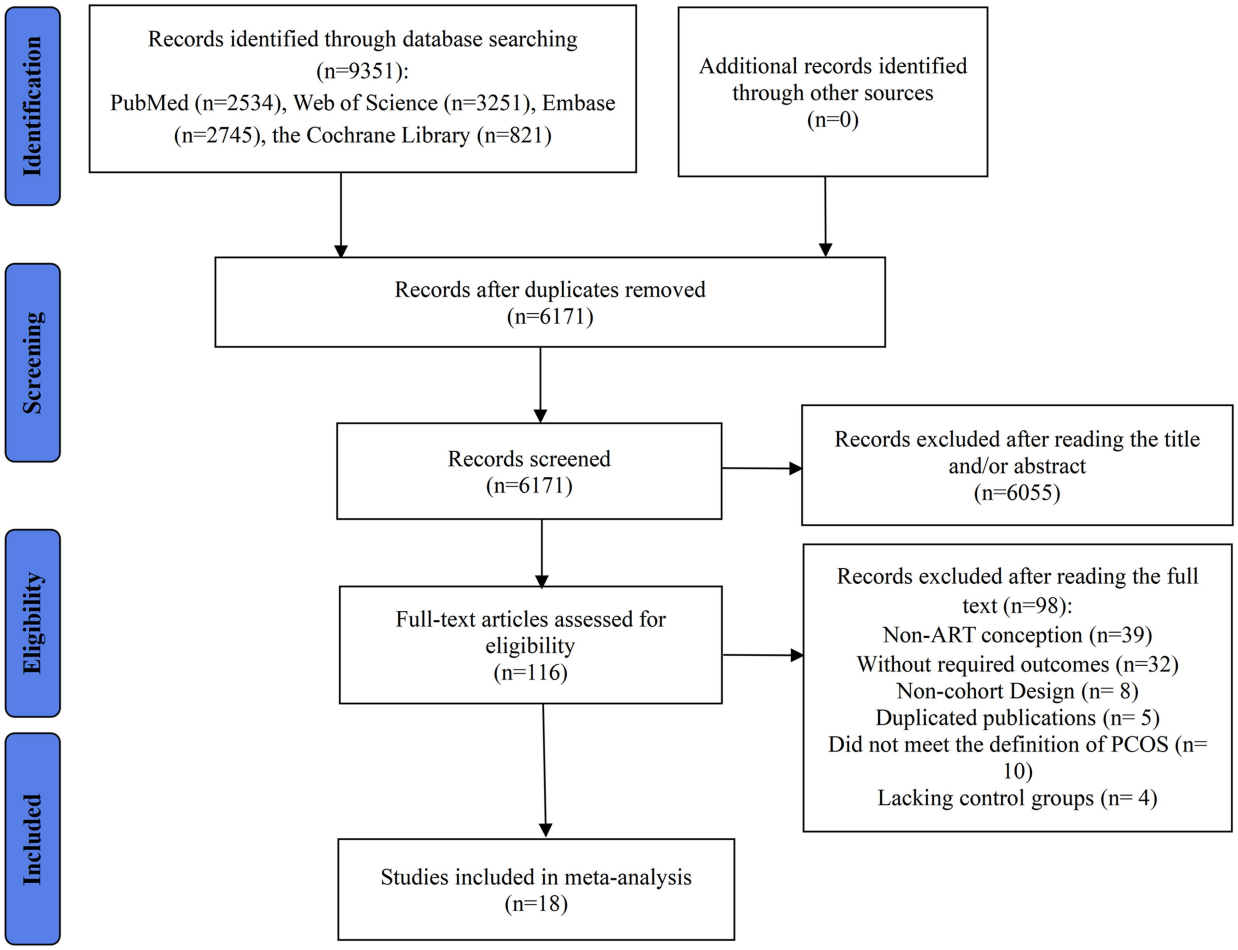
Flow diagram of the process of study selection.

A systematic database search initially identified 9,351 articles for potential inclusion. Following the removal of 3,180 duplicate records using EndNote X9 software and the exclusion of 6,055 studies based on a preliminary screening of titles and abstracts, 116 articles were retained for detailed evaluation. After applying the inclusion and exclusion criteria, 98 studies were excluded from the final meta-analysis. Of these, 39 studies were removed because not all patients received ART conception, and 32 were excluded due to the absence of reported RRs or ORs with corresponding 95% CIs for adverse pregnancy or perinatal outcomes. Additionally, 8 studies were excluded due to their case–control or cross-sectional design, and 5 were removed as overlapping cohorts. A further 10 studies were excluded for failing to meet the definition of the PCOS group, and 4 lacked an appropriate control group. Ultimately, 18 studies met the eligibility criteria and were included in the meta-analysis (9, 11, 12, 24–38) (Figure 1).

### 3.2 Characteristics and quality assessment of the included studies

The characteristics of the included studies were summarized in Table 1. To ensure relevance and timeliness, only research published from 2009 onwards was eligible for inclusion in the meta-analysis. A total of 18 retrospective cohort studies were analyzed, comprising 16,365 participants diagnosed with PCOS and 111,503 individuals in the control group. The maternal outcomes assessed in the meta-analysis included clinical pregnancy rate, miscarriage, GDM, hypertensive disorders of pregnancy (HDP), gestational hypertension, PPROM, and cesarean delivery. Fetal outcomes comprised live birth rate, PTB, VPTB, low birth weight (LBW), very low birth weight (VLBW), macrosomia, small for gestational age (SGA), very small for gestational age (VSGA), LGA, and fetal malformation. According to the World Health Organization (WHO), PTB was defined as delivery before 37 weeks of gestation, while VPTB referred to delivery before 32 weeks. LBW and VLBW were categorized as birth weights below 2,500 g and 1,500 g, respectively, whereas macrosomia was defined as a birth weight exceeding 4,000 g. SGA and VSGA were classified as birth weights below the 10th and 3rd percentiles, respectively (39), while LGA was defined as a birth weight above the 90th percentile. All studies included in the meta-analysis were deemed to be of high methodological quality, as they provided comprehensive descriptions of their study designs (Supplemental File 2).

**Table 1:** Characteristics of included studies.

Study ID	Country	Design	Sample size (E/C)	Diagnosis of PCOS	Controls	PCOS		Control		Mode of ART	Adjusted confounders	Outcomes
						Age (years)	BMI (kg/m^2^)	Age (years)	BMI (kg/m^2^)			
Lin et al. (2021) (9)	China	RCS	1,167/9,995	2003 Rotterdam criteria	Patients with tubal factor infertility or male factor infertility	All age groups	≤29.9	All age groups	≤29.9	Frozen ET	Maternal age, primary infertility, parity, infertility duration, type of ART procedure, number of embryos transferred, embryo stage at transfer, offspring gender, year of birth, and maternal BMI	9, 10, 11, 13, 14, 16
Qiu et al. (2022) (12)	China	RCS	1,876/14,630	2003 Rotterdam criteria	Patients with tubal factor infertility or male factor infertility	<38	All BMI groups	<38	All BMI groups	Frozen ET	Maternal age, maternal BMI, infertility, parity, FET cycle rank, insemination method, embryo stage, number of embryos transferred, FET endometrial preparation, mode of delivery, GDM, pregnancy-induced hypertension, pre-eclampsia, abnormal placentation and PPROM	3, 5, 6, 7, 9, 10, 11, 12, 13, 14, 15, 16
Sterling et al. (2016) (11)	Canada	RCS	173/911	2003 Rotterdam criteria	Non-PCOS	Median (IQR): 33 (30–35)	Median (IQR): 22.7 (20.4–28.3)	Median (IQR): 35 (32–37)	Median (IQR): 22.6 (20.8–26.0)	Fresh ET	Maternal age, BMI, parity with or without time to conception	3, 4, 6, 7, 9, 10, 11, 12, 13, 14, 16, 17
Aihaiti et al. (2024) (24)	China	RCS	693/2,262	2003 Rotterdam criteria	Non-PCOS	30.02 ± 3.49	23.30 ± 3.42	31.20 ± 3.83	21.84 ± 2.68	Frozen ET	Maternal age, BMI, infertility duration, fertilization method, and multiple pregnancies	3, 5, 6, 9, 11, 13, 14, 16, 17
Beydoun et al. (2009) (25)	USA	RCS	69/69	1990 National Institute of Health (NIH) criteria	Non-PCOS	32.30 ± 4.11	30.60 ± 8.94	32.49 ± 4.08	23.91 ± 4.86	NR	Age, BMI, day 3 follicle-stimulating hormone, day 3 luteinizing hormone, total follicle-stimulating hormone dosage, and total luteinizing hormone dosage	1, 2, 8
Zhang et al. (2023) (38)	China	RCS	156/344	2003 Rotterdam criteria	Patients with infertility with only tubal factor	Median (IQR): 37.0 (36.0–39.0)	25.52 ± 3.38	Median (IQR): 37.0 (35.0–39.0)	22.30 ± 2.31	Fresh/Frozen ET	NR	1, 8
Wang et al. (2022) (37)	China	RCS	1,186/5,546	2003 Rotterdam criteria	Non-PCOS	≤38	All BMI groups	≤38	All BMI groups	Fresh ET	NR	4
Luo et al. (2017) (35)	China	RCS	67/201	2003 Rotterdam criteria	Non-PCOS	30.3 ± 3.1	21.3 ± 2.2	30.5 ± 4.3	21.5 ± 2.1	Fresh ET	Age, BMI, previous early miscarriage, endometrium thickness	2, 8
Cai et al. (2021) (26)	China	RCS	2,357/19,463	2003 Rotterdam criteria	Non-PCOS	29.0 ± 3.3	24.3 ± 3.7	30.5 ± 4.2	22.3 ± 3.2	Fresh/Frozen ET	Maternal age, overweight/BMI, history of spontaneous miscarriage, number of embryos transferred and medical conditions (diabetes, hypertensive disease)	8
Hu et al. (2021) (29)	China	RCS	557/3,526	2003 Rotterdam criteria	Non-PCOS	29.67 ± 3.57	22.63 ± 3.24	31.56 ± 4.17	21.53 ± 2.83	Frozen ET	Age, BMI, ICSI, D5 blastocyst, primary infertility, infertility years, male factors, pelvic and tubal factors, and the number of oocytes retrieved	1, 3, 4, 7, 8, 9, 11, 13, 17
Hu et al. (2024) (30)	China	RCS	1,667/12,256	2003 Rotterdam criteria	Non-PCOS	NR	All BMI groups	NR	All BMI groups	Frozen ET	Age, number of embryos transferred, stage of embryo development, endometrial preparation protocol, fertilization method, cause of infertility, endometrial thickness, and number of oocytes retrieved	1, 3, 4, 8, 9, 10, 11, 12, 14, 15, 16
Li et al. (2024) (32)	China	RCS	206/360	2003 Rotterdam criteria	Patients with infertility with only tubal factor	<35	All BMI groups	<35	All BMI groups	Fresh/Frozen ET	NR	1
Liu et al. (2020) (33)	China	RCS	666/7,012	2003 Rotterdam criteria	Non-PCOS	Median (IQR): 30.0 (27.0–32.0)	Median (IQR): 22.3 (20.3–25.0)	Median (IQR): 31.0 (29.0–34.0)	Median (IQR): 20.7 (19.2–22.6)	Fresh ET	Maternal age, BMI, infertility duration, total dose of gonadotropin, serum E2 level and endometrial thickness on hCG day, number of fertilized occytes, number of embryos transferred, embryo type and embryo quality	1, 2, 5, 8, 9, 10,
Dou et al. (2023) (27)	China	RCS	613/2,363	2003 Rotterdam criteria	Non-PCOS	29.19 ± 4.01	Median (IQR): 24.40 (22.00–27.30)	31.59 ± 4.67	Median (IQR): 22.60 (20.60–25.10)	Fresh ET	None	2, 8
Liu et al. (2024) (34)	China	RCS	787/4,052	2003 Rotterdam criteria	Patients who underwent first IVF/ICSI treatment due to fallopian tubal factor or male factor infertility	29.39 ± 3.53	22.60 ± 3.43	31.97 ± 4.28	21.50 ± 2.81	Fresh/Frozen ET	NR	2
Jie et al. (2022) (31)	China	RCS	336/2,325	2003 Rotterdam criteria	Patients who underwent IVF/ICSI due to fallopian tubal factor or male factor infertility	Median: 30	Median: 21.6	Median: 32	Median: 20.8	Frozen ET	None	8, 9
Wang, Zheng et al. (2022) (37)	China	RCS	1,887/7,016	2003 Rotterdam criteria	Non-PCOS	20–40	≤35	30.97 ± 4.14	23.38 ± 3.33	Frozen ET	NR	2, 9
Guo et al. (2025) (28)	China	RCS	1,902/19,172	NR	Non-PCOS	Median (IQR): 29.0 (27.0–31.0)	Median (IQR): 22.58 (20.40–25.00)	Median (IQR): 32.0 (29.0–36.0)	Median (IQR): 21.36 (19.68–23.44)	Frozen ET	Maternal age at FET, duration of infertility, infertility diagnosis, fertilization, No. of embryos transferred, type of embryos transferred	1, 2, 3, 4, 8, 9, 10, 11, 12, 13, 14, 15, 16, 17

E, exposure group; C, control group; RCS, retrospective cohort study; PCOS, polycystic ovary syndrome; ART, assisted reproductive technology; ET, embryo transfer; IQR, interquartile range; NR, not reported; IVF, in vitro fertilization; ICSI, intracytoplasmic sperm injection; 1, clinical pregnancy rate; 2, miscarriage; 3, gestational diabetes mellitus; 4, hypertensive disorders of pregnancy; 5, gestational hypertension; 6, preterm premature rupture of membranes; 7, cesarean delivery; 8, live birth rate; 9, preterm birth; 10, very preterm birth; 11, low birth weight; 12, very low birth weight; 13, macrosomia; 14, small for gestational age; 15, very small for gestational age; 16, large for gestational age; 17, fetal malformation.

### 3.3 Meta-analysis of adverse maternal outcomes

#### 3.3.1 Clinical pregnancy rate and miscarriage

**Figure 2:**
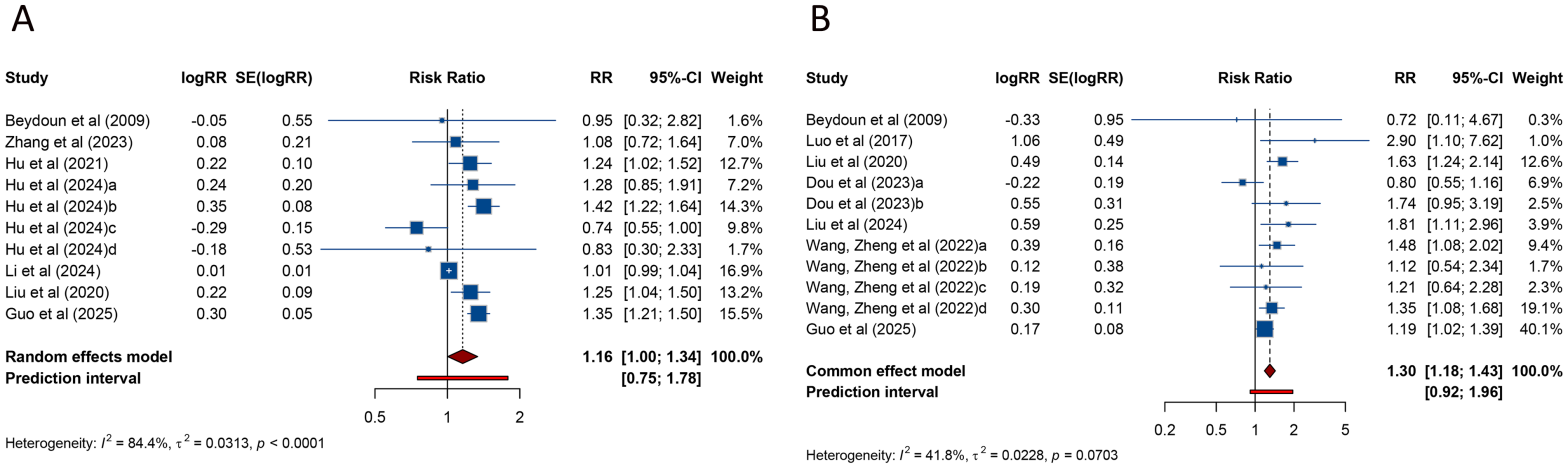
Forest plots of the association between polycystic ovary syndrome and clinical pregnancy rate **(A)** and miscarriage **(B)** in women undergoing assisted reproductive technology.

The meta-analysis of 10 studies investigating the clinical pregnancy rate in women with PCOS undergoing ART revealed a pooled RR of 1.158 (95% CI: 1.004–1.335; 95% PI: 0.751–1.785), indicating a modest increase in clinical pregnancy rates compared with women without PCOS. Notably, substantial heterogeneity was detected across the included studies (*I*^2^ = 84.4%, Tau^2^ = 0.0313) (Table 2; Figure 2A). Subgroup analysis revealed that the association between PCOS and higher clinical pregnancy rate persisted in women receiving FET (RR = 1.204, 95% CI: 1.018–1.423; 95% PI: 0.760–1.906), though significant heterogeneity was still present (*I*^2^ = 68.9%, Tau^2^ = 0.0246). However, whereas no significant association was observed among fresh/frozen embryo transfer patients (RR = 1.015, 95% CI: 0.993–1.037; 95% PI: 0.882–1.168) (Table 2; Supplementary Figure S1A,B).

**Table 2:** Pooled effect and subgroup analysis of the association between polycystic ovary syndrome and adverse maternal outcomes in women who had undergone ART.

Outcomes and subgroups	Number of studies	Meta-analysis				Heterogeneity	
		RR	95% CI	*p* value	95% PI	*I*^2^, Tau^2^	*p* value
Clinical pregnancy rate	10	1.158	1.004–1.335	0.045	0.751–1.785	84.4%, 0.0313	<0.001
Fresh/Frozen ET	2	1.015	0.993–1.037	0.173	0.882–1.168	0%, 0	0.759
Frozen ET	6	1.204	1.018–1.423	0.030	0.760–1.906	68.9%, 0.0246	0.007
Miscarriage	11	1.301	1.181–1.433	<0.001	0.917–1.957	41.8%, 0.0228	0.070
Fresh ET	4	1.458	0.897–2.369	0.128	0.315–6.755	76.2%, 0.1707	0.006
Frozen ET	5	1.263	1.127–1.415	<0.001	1.075–1.483	0%, 0	0.739
GDM	8	1.456	1.137–1.864	0.003	0.739–2.867	67.7%, 0.0662	0.003
Frozen ET	7	1.383	1.085–1.762	0.009	0.716–2.671	67.2%, 0.0571	0.006
HDP	10	1.523	1.218–1.905	<0.001	0.801–2.995	29.0%, 0.0613	0.178
Fresh ET	5	2.203	1.323–3.667	0.002	0.520–8.753	27.8%, 0.1495	0.236
Frozen ET	5	1.395	1.088–1.789	0.009	0.813–2.327	13.8%, 0.0151	0.326
Gestational hypertension	3	1.470	1.226–1.762	<0.001	0.986–2.190	0%, 0	0.806
Frozen ET	2	1.442	1.191–1.745	<0.001	0.418–4.973	0%, 0	0.887
PPROM	3	1.532	1.225–1.916	<0.001	0.937–2.503	0%, 0	0.528
Frozen ET	2	1.509	1.202–1.895	<0.001	0.345–6.597	0%, 0	0.373
Cesarean delivery	3	0.898	0.810–0.994	0.039	0.717–1.124	0%, 0	0.482
Frozen ET	2	0.894	0.806–0.993	0.036	0.181–4.667	24.8%, 0.0076	0.249

ART, assisted reproductive technology; ET, embryo transfer; GDM, gestational diabetes mellitus; HDP, hypertensive disorders of pregnancy; PPROM, preterm premature rupture of membranes.

Regarding miscarriage, a meta-analysis of 11 studies indicated that PCOS was associated with a significantly higher risk of miscarriage (RR = 1.301, 95% CI: 1.181–1.433; 95% PI: 0.917–1.957), with no significant heterogeneity (*I*^2^ = 41.8%, Tau^2^ = 0.0228) (Table 2; Figure 2B). Subgroup analysis revealed that the heightened miscarriage risk was significant in patients undergoing FET (RR = 1.263, 95% CI: 1.127–1.415; 95% PI: 1.075–1.483), whereas no such association was found in those receiving fresh embryo transfer (RR = 1.458, 95% CI: 0.897–2.369; 95% PI: 0.315–6.755) (Table 2; Supplementary Figure S1C,D).

#### 3.3.2 GDM, HDP, and gestational hypertension

**Figure 3:**
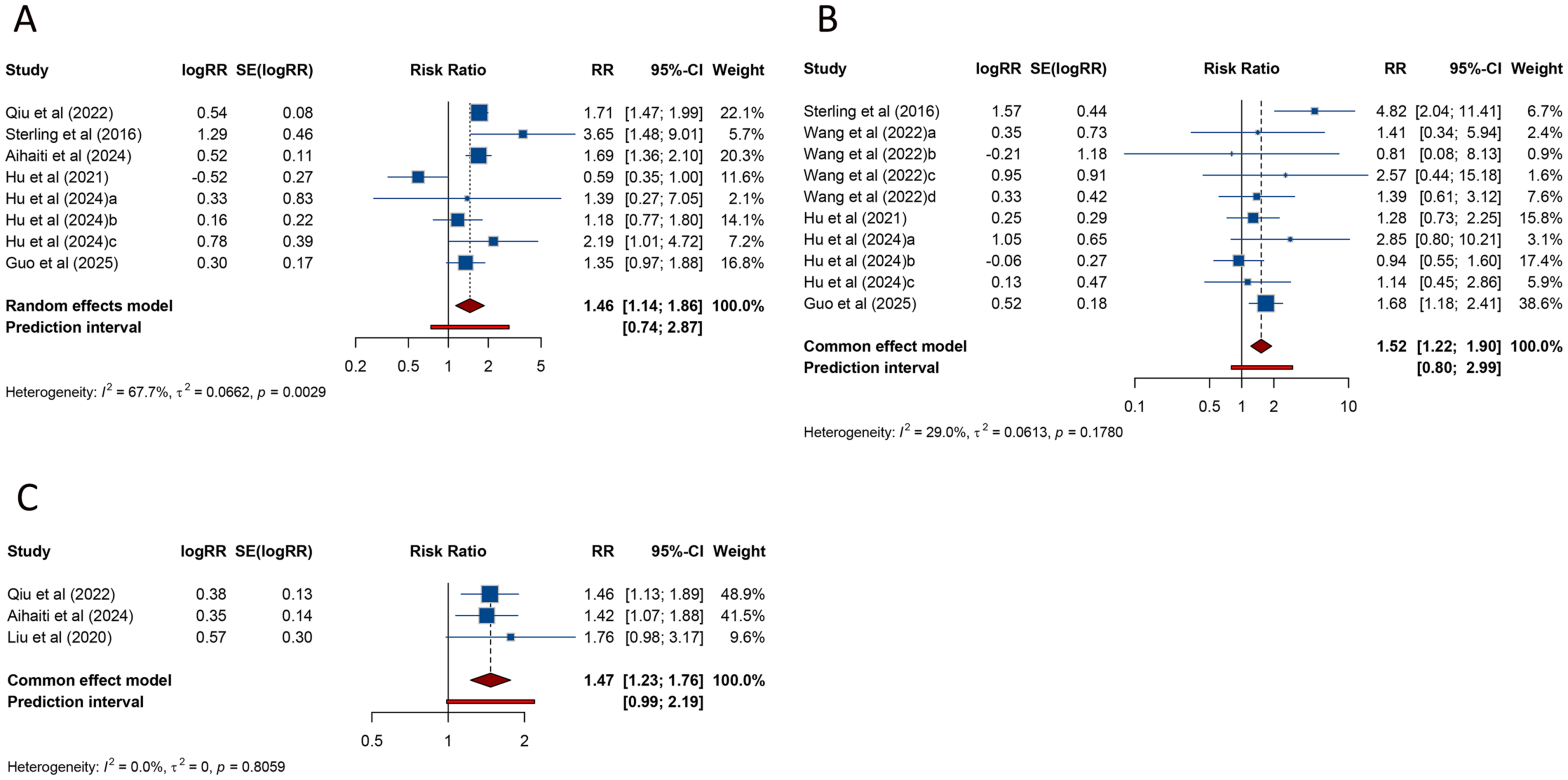
Forest plots of the association between polycystic ovary syndrome and gestational diabetes mellitus **(A)**, hypertensive disorders of pregnancy **(B)**, and gestational hypertension **(C)** in women undergoing assisted reproductive technology.

Eight studies assessed the risk of GDM in women with PCOS undergoing ART. The combined analysis demonstrated that PCOS patients had a significantly increased risk of GDM compared to those without PCOS (RR = 1.456, 95% CI: 1.137–1.864; 95% PI: 0.739–2.867), accompanied by notable heterogeneity across studies (*I*^2^ = 67.7%, Tau^2^ = 0.0662) (Table 2; Figure 3A). Subgroup analysis showed that this elevated risk remained significant in patients undergoing FET (RR = 1.383, 95% CI: 1.085–1.762; 95% PI: 0.716–2.671) (Table 2; Supplementary Figure S2A).

HDP was examined in 10 studies, with pooled data indicating a significantly higher risk in women with PCOS compared to controls (RR = 1.523, 95% CI: 1.218–1.905; 95% PI: 0.801–2.995). Unlike the findings for GDM, no significant heterogeneity was observed (*I*^2^ = 29.0%, Tau^2^ = 0.0613) (Table 2; Figure 3B). Subgroup analysis confirmed that this association persisted in women undergoing either fresh embryo transfer (RR = 2.203, 95% CI: 1.323–3.667; 95% PI: 0.520–8.753) or FET (RR = 1.395, 95% CI: 1.088–1.789; 95% PI: 0.813–2.327) (Table 2; Supplementary Figure S2B,C).

Three studies specifically addressed gestational hypertension, showing a significantly increased risk in women with PCOS (RR = 1.470, 95% CI: 1.226–1.762; 95% PI: 0.986–2.190), with no evidence of heterogeneity (*I*^2^ = 0%, Tau^2^ = 0) (Table 2; Figure 3C). This elevated risk was also observed in the subgroup of patients undergoing FET (RR = 1.442, 95% CI: 1.191–1.745; 95% PI: 0.418–4.973) (Table 2; Supplementary Figure S2D).

#### 3.3.3 PPROM and cesarean delivery

**Figure 4:**
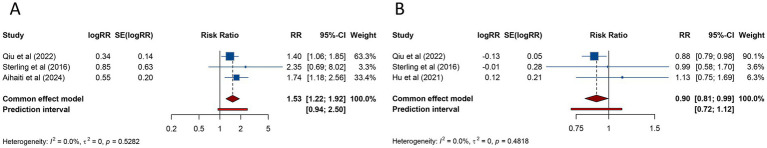
Forest plots of the association between polycystic ovary syndrome and preterm premature rupture of membranes **(A)** and cesarean delivery **(B)** in women undergoing assisted reproductive technology.

Three studies assessed the risk of PPROM in women with PCOS undergoing ART. The pooled analysis identified a significantly elevated risk of PPROM in patients with PCOS compared to those without the condition (RR = 1.532, 95% CI: 1.225–1.916; 95% PI: 0.937–2.503), with no evidence of substantial heterogeneity (*I*^2^ = 0%, Tau^2^ = 0) (Table 2; Figure 4A). Subgroup analysis further revealed that the increased risk of PPROM persisted among women undergoing FET (RR = 1.509, 95% CI: 1.202–1.895; 95% PI: 0.345–6.597) (Table 2; Supplementary Figure S3A).

Cesarean delivery was investigated in 3 studies, with pooled findings showing a significantly reduced likelihood of cesarean delivery among women with PCOS (RR = 0.898, 95% CI: 0.810–0.994; 95% PI: 0.717–1.124). No substantial heterogeneity was detected in the analysis (*I*^2^ = 0%, Tau^2^ = 0) (Table 2; Figure 4B). Subgroup analysis also demonstrated that the lower risk of cesarean delivery persisted in women who underwent FET (RR = 0.894, 95% CI: 0.806–0.993; 95% PI: 0.181–4.667) (Table 2; Supplementary Figure S3B).

### 3.4 Meta-analysis of adverse fetal outcomes

#### 3.4.1 Live birth rate, PTB, and VPTB

**Figure 5:**
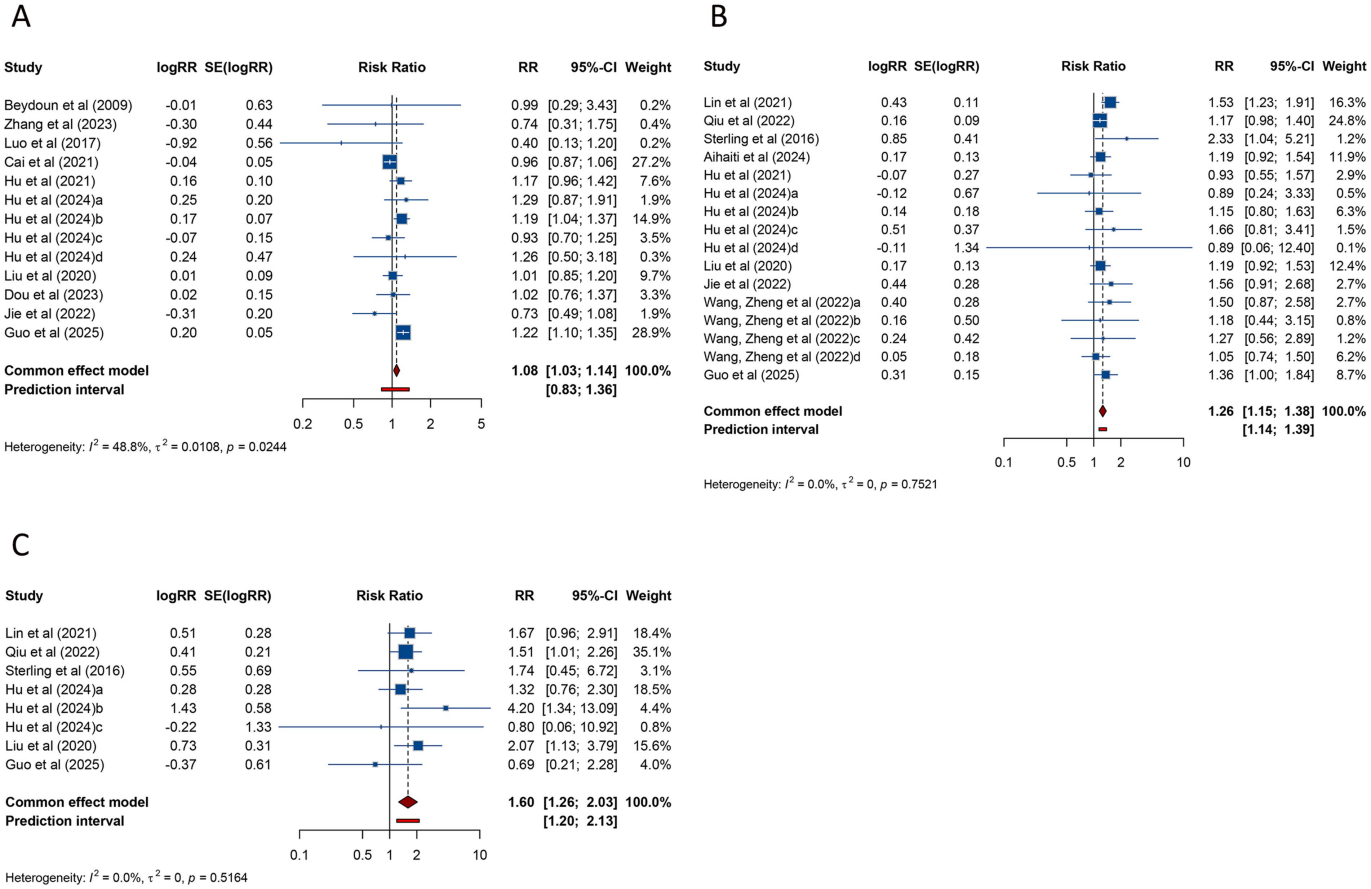
Forest plots of the association between polycystic ovary syndrome and live birth rate **(A)**, preterm birth **(B)**, and very preterm birth **(C)** in women undergoing assisted reproductive technology.

The meta-analysis of 13 studies evaluating live birth rate in women with PCOS undergoing ART revealed a pooled RR of 1.084 (95% CI: 1.027–1.144; 95% PI: 0.827–1.361), suggesting a modest increase in live birth rate compared to women without PCOS. No significant heterogeneity was observed (*I*^2^ = 48.8%, Tau^2^ = 0.0108) (Table 3; Figure 5A). Subgroup analysis indicated that the association between PCOS and higher live birth rate was significant in patients undergoing FET (RR = 1.171, 95% CI: 1.092–1.256; 95% PI: 0.918–1.421), but not in those receiving fresh embryo transfer or combined fresh/frozen embryo transfer (all *p* > 0.05) (Table 3; Supplementary Figure S4A–C).

**Table 3:** Pooled effect and subgroup analysis of the association between polycystic ovary syndrome and adverse fetal outcomes in women who had undergone ART.

Outcomes and subgroups	Number of studies	Meta-analysis				Heterogeneity	
		RR	95% CI	*p* value	95% PI	*I*^2^, Tau^2^	*p* value
Live birth rate	13	1.084	1.027–1.144	0.004	0.827–1.361	48.8%, 0.0108	0.024
Fresh/Frozen ET	2	0.957	0.863–1.060	0.396	0.491–1.862	0%, 0	0.560
Fresh ET	3	0.997	0.860–1.156	0.968	0.524–1.843	26.1%, 0.0101	0.259
Frozen ET	7	1.171	1.092–1.256	<0.001	0.918–1.421	31.8%, 0.0053	0.186
PTB	16	1.259	1.152–1.376	<0.001	1.143–1.387	0%, 0	0.752
Fresh ET	2	1.485	0.797–2.769	0.213	0.003–708.499	59.2%, 0.1346	0.117
Frozen ET	14	1.259	1.144–1.385	<0.001	1.133–1.399	0%, 0	0.806
VPTB	8	1.597	1.258–2.027	<0.001	1.198–2.130	0%, 0	0.516
Fresh ET	2	2.013	1.160–3.493	0.013	0.056–71.796	0%, 0	0.817
Frozen ET	6	1.514	1.162–1.973	0.002	0.980–2.343	6.0%, 0.0082	0.379
LBW	10	1.082	0.942–1.242	0.266	0.631–1.696	38.2%, 0.0362	0.104
Frozen ET	9	1.075	0.935–1.236	0.311	0.588–1.756	44.1%, 0.0429	0.074
VLBW	5	1.476	0.971–2.245	0.069	0.815–2.674	0%, 0	0.531
Frozen ET	4	1.470	0.945–2.286	0.088	0.604–3.617	5.0%, 0.0183	0.368
Macrosomia	6	0.979	0.770–1.243	0.859	0.499–1.921	67.2%, 0.0539	0.009
Frozen ET	5	0.979	0.757–1.266	0.872	0.452–2.124	73.6%, 0.0606	0.004
SGA	8	0.973	0.835–1.134	0.727	0.645–1.543	29.7%, 0.0234	0.191
Frozen ET	7	0.973	0.833–1.136	0.727	0.597–1.689	39.8%, 0.0324	0.126
VSGA	5	1.110	0.805–1.532	0.525	0.704–1.752	0%, 0	0.514
Frozen ET	5	1.110	0.805–1.532	0.525	0.704–1.752	0%, 0	0.514
LGA	9	1.044	0.892–1.221	0.594	0.693–1.571	56.7%, 0.0250	0.018
Frozen ET	8	1.015	0.877–1.174	0.843	0.702–1.466	52.4%, 0.0187	0.040
Fetal malformation	4	1.218	0.835–1.778	0.307	0.659–2.251	0%, 0	0.586
Frozen ET	3	1.241	0.840–1.833	0.278	0.527–2.921	0%, 0	0.408

ART, assisted reproductive technology; ET, embryo transfer; PTB, preterm birth; VPTB, very preterm birth; LBW, low birth weight; VLBW, very low birth weight; SGA, small for gestational age; VSGA, very small for gestational age; LGA, large for gestational age.

PTB, analyzed across 16 studies, was found to be significantly more frequent in women with PCOS undergoing ART (RR = 1.259, 95% CI: 1.152–1.376; 95% PI: 1.143–1.387), with no evidence of heterogeneity (*I*^2^ = 0%, Tau^2^ = 0) (Table 3; Figure 5B). Subgroup analysis revealed that the increased risk of PTB was significant in patients undergoing FET (RR = 1.259, 95% CI: 1.144–1.385; 95% PI: 1.133–1.399) but not in those receiving fresh embryo transfer (RR = 1.485, 95% CI: 0.797–2.769; 95% PI: 0.003–708.499) (Table 3; Supplementary Figure S5A,B).

The risk of VPTB was examined in 8 studies, with pooled results showing a significantly higher incidence in women with PCOS (RR = 1.597, 95% CI: 1.258–2.027; 95% PI: 1.198–2.130). No heterogeneity was detected (*I*^2^ = 0%, Tau^2^ = 0) (Table 3; Figure 5C). Subgroup analysis confirmed that this elevated risk was consistent across fresh embryo transfer (RR = 2.013, 95% CI: 1.160–3.493; 95% PI: 0.056–71.796) or FET (RR = 1.514, 95% CI: 1.162–1.973; 95% PI: 0.980–2.343) (Table 3; Supplementary Figure S5C,D).

#### 3.4.2 LBW, VLBW, and macrosomia

**Figure 6:**
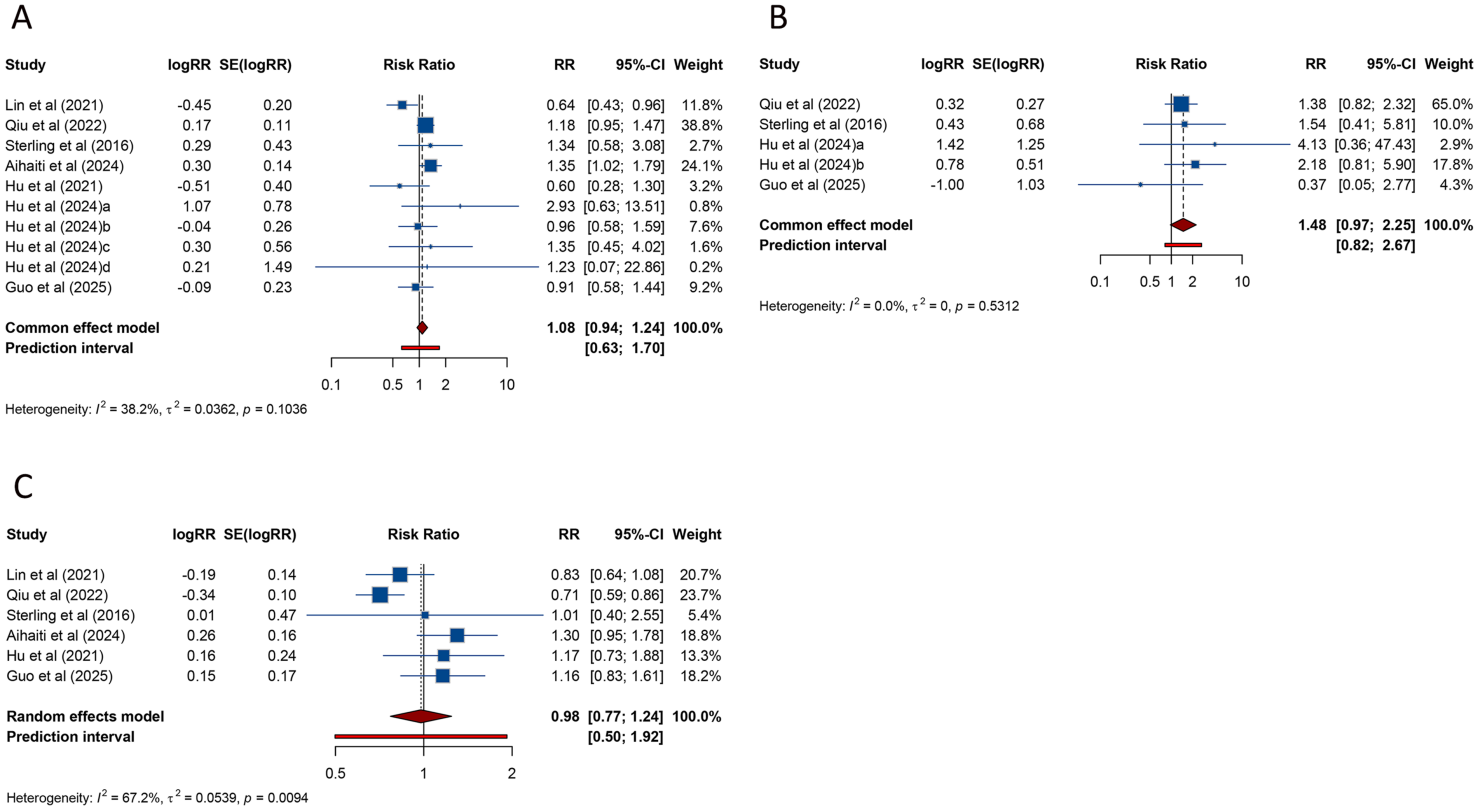
Forest plots of the association between polycystic ovary syndrome and low birth weight **(A)**, very low birth weight **(B)**, and macrosomia **(C)** in women undergoing assisted reproductive technology.

The risk of LBW was assessed in 10 studies, with pooled analysis showing no significant association between PCOS and LBW (RR = 1.082, 95% CI: 0.942–1.242; 95% PI: 0.631–1.696), although heterogeneity was not significant (*I*^2^ = 38.2%, Tau^2^ = 0.0362) (Table 3; Figure 6A). Subgroup analysis focusing on FET similarly found no evidence of a significant association (RR = 1.075, 95% CI: 0.935–1.236; 95% PI: 0.588–1.756) (Table 3; Supplementary Figure S6A).

VLBW was investigated in 5 studies, and the pooled findings indicated no substantial increase in risk for women with PCOS undergoing ART (RR = 1.476, 95% CI: 0.971–2.245; 95% PI: 0.815–2.674) (Table 3; Figure 6B). Subgroup analysis for FET also demonstrated no significant association (RR = 1.470, 95% CI: 0.945–2.286; 95% PI: 0.604–3.617) (Table 3; Supplementary Figure S6B).

Macrosomia, assessed in 6 studies, showed no significant association with PCOS (RR = 0.979, 95% CI: 0.770–1.243; 95% PI: 0.499–1.921), with significant heterogeneity (*I*^2^ = 67.2%, Tau^2^ = 0.0539) (Table 3; Figure 6C). Subgroup analysis for FET similarly showed no significant association (RR = 0.979, 95% CI: 0.757–1.266; 95% PI: 0.452–2.124) (Table 3; Supplementary Figure S6C).

#### 3.4.3 SGA, VSGA, LGA, and fetal malformation

**Figure 7:**
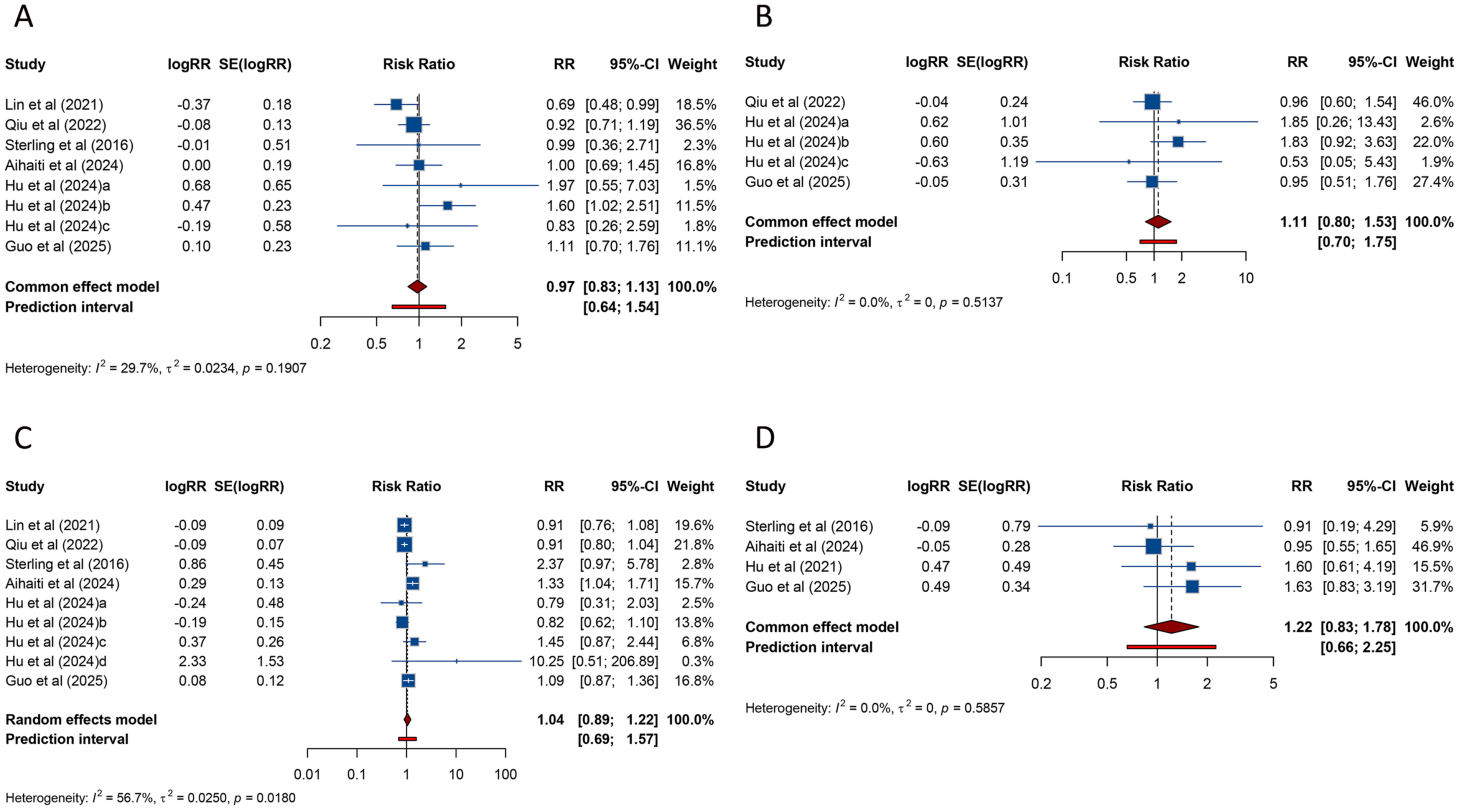
Forest plots of the association between polycystic ovary syndrome and small for gestational age **(A)**, very small for gestational age **(B)**, large for gestational age **(C)**, and fetal malformation **(D)** in women undergoing assisted reproductive technology.

The relationship between PCOS and SGA was investigated in 8 studies, with pooled data indicating no significant association (RR = 0.973, 95% CI: 0.835–1.134; 95% PI: 0.645–1.543) and an absence of heterogeneity (*I*^2^ = 29.7%, Tau^2^ = 0.0234) (Table 3; Figure 7A). Subgroup analysis of FET yielded comparable results (RR = 0.973, 95% CI: 0.833–1.136; 95% PI: 0.597–1.689) (Table 3; Supplementary Figure S7A).

VSGA was analyzed in 5 studies, which similarly found no significant association between PCOS and VSGA (RR = 1.110, 95% CI: 0.805–1.532; 95% PI: 0.704–1.752), with heterogeneity remaining negligible (*I*^2^ = 0%, Tau^2^ = 0) (Table 3; Figure 7B). Consistent results were obtained among women receiving FET (*p* < 0.05) (Table 3; Supplementary Figure S7B).

Nine studies examined LGA among women undergoing ART. Pooled analysis demonstrated no significant link between PCOS and LGA (RR = 1.044, 95% CI: 0.892–1.221; 95% PI: 0.693–1.571) (Table 3; Figure 7C), and subgroup analysis focusing on FET showed consistent trends (RR = 1.015, 95% CI: 0.877–1.174; 95% PI: 0.702–1.466) (Table 3; Supplementary Figure S7C).

Four studies reported fetal malformation. The meta-analysis revealed no significant association between PCOS and fetal malformation (RR = 1.218, 95% CI: 0.835–1.778; 95% PI: 0.659–2.251), with no significant heterogeneity (*I*^2^ = 0%, Tau^2^ = 0) (Table 3; Figure 7D). Subgroup analysis for FET showed similar findings (RR = 1.241, 95% CI: 0.840–1.833; 95% PI: 0.527–2.921) (Table 3; Supplementary Figure S7D).

### 3.5 Sensitivity analysis and publication bias

Sensitivity analyses and tests for publication bias were conducted solely for maternal and fetal outcomes that included 10 or more studies. To evaluate the robustness of the results, a leave-one-out method was applied, which demonstrated that excluding any individual study had no notable impact on the overall conclusions regarding miscarriage, HDP, and PTB. These findings highlight the stability and reliability of our results (Supplementary Figure S8). Publication bias was examined using Begg’s and Egger’s tests, neither of which identified significant bias among the included studies (all *p* > 0.05). Corresponding funnel plots were presented in Supplementary Figure S9.

## 4 Discussion

This study utilized cohort studies to explore the potential causal associations between PCOS and adverse pregnancy and perinatal outcomes among women undergoing ART. Our meta-analysis revealed that compared with non-PCOS patients undergoing ART, women with PCOS who had undergone ART showed higher clinical pregnancy rate and live birth rate. Nonetheless, they were also found to have significantly elevated risks of miscarriage, GDM, HDP, gestational hypertension, PPROM, PTB, and VPTB. Conversely, the likelihood of cesarean delivery was lower in PCOS patients. No significant differences were identified between PCOS and non-PCOS groups in the risks of LBW, VLBW, macrosomia, SGA, VSGA, LGA, or fetal malformation. Further subgroup analyses demonstrated consistent statistical significance among women who conceived through FET.

The clinical pregnancy rate and live birth rate, which integrate outcomes from both fresh embryo transfer and FET cycles, offer a robust measure of the overall effectiveness of IVF/ICSI procedures (40, 41). Research by Liu et al. reported that women with PCOS had significantly greater numbers of oocytes retrieved and fertilized. This enhanced ovarian response facilitates the collection of more oocytes and improves fertilization potential, allowing for better embryo selection and ultimately contributing to higher pregnancy rates (33). The ovaries of women with PCOS harbor a higher follicular reserve compared to those without the condition (42), and biomarkers such as elevated serum anti-Müllerian hormone (AMH) levels and increased antral follicle counts (AFC) remain consistently high, even beyond the age of 35 years (43–45). This abundant ovarian reserve provides the foundation for generating a sufficient number of embryos, which underpins the observed improvements in clinical pregnancy rate and live birth rate. However, subgroup analyses in this study revealed that the observed improvements in clinical pregnancy rate and live birth rate were confined to patients undergoing FET. No statistically significant differences were observed among those undergoing fresh embryo transfer or mixed (fresh/frozen) embryo transfer. These results suggest potential advantages of FET for women with PCOS. By decoupling ovarian stimulation from embryo transfer, the FET approach offers an opportunity to mitigate the risks associated with ovarian hyperstimulation syndrome (OHSS) and allows time for the endometrium to recover (46), fostering a more receptive environment for implantation. Additionally, FET circumvents the adverse impact of elevated estrogen levels on endometrial receptivity, a common occurrence during fresh embryo transfer cycles (47). Women with PCOS, due to their heightened ovarian sensitivity to gonadotropins, are more prone to excessive estrogen production during fresh cycles (48, 49), which may impair endometrial receptivity and compromise embryo implantation and pregnancy maintenance (50).

Several meta-analyses have systematically explored the reproductive outcomes of IVF and ICSI in women with PCOS, consistently demonstrating a higher risk of miscarriage compared with women without PCOS (8, 13, 51). Retrospective data from 2,357 women with PCOS who conceived via IVF revealed a significantly increased incidence of late miscarriage (26). Notably, even after accounting for chromosomal abnormalities in embryos, the miscarriage rate among PCOS patients remained substantially higher than that of the control group (31). Our pooled analysis corroborated these findings, identifying an elevated risk of miscarriage in women with PCOS undergoing ART compared with non-PCOS counterparts. Recent research suggests that the elevated miscarriage risk associated with PCOS may be linked to factors such as hyperandrogenism and insulin resistance, which can interfere with mitochondrial function and disrupt the balance between oxidative stress and antioxidant defense mechanisms in the uterus during pregnancy (52). However, it is important to note that these findings are based on animal studies, and their applicability to human cases has yet to be confirmed. In our subgroup analysis, we observed that women with PCOS faced a markedly higher miscarriage risk following FET, whereas no similar increase was identified after fresh embryo transfer. These findings highlight the potential role of FET as a contributing factor to miscarriage in patients with PCOS. The FET procedure involves freezing and thawing embryos, which could potentially affect embryo viability and contribute to the observed rise in miscarriage risk. Furthermore, although FET is often associated with a more natural hormonal environment for endometrial preparation, the underlying pathological alterations in the endometrium of women with PCOS, such as chronic inflammation and abnormal angiogenesis (53, 54), may persist, thereby limiting the potential benefits of FET.

PTB remains a major contributor to neonatal mortality and morbidity (55). Numerous studies have consistently shown that women PCOS face a substantially higher risk of PTB and VPTB following ART (11, 12, 28). Our findings further support this association. Chronic low-grade inflammation and hyperandrogenism, characteristic of PCOS, are hypothesized to compromise placental development and perfusion (56), leading to placental dysfunction and a subsequent increase in PTB risk. An interaction between PCOS and ART appears to further compound the risk of PTB, as many women with PCOS rely on ART to conceive. Importantly, pregnancies achieved through ART are independently associated with an increased likelihood of PTB, even in singleton gestations (57). A retrospective cohort study by Naver et al. (58) similarly identified an increased incidence of PTB in women with PCOS compared with the general population, based on logistic regression analyses adjusted for maternal age, BMI, and parity. However, as this study included pregnancies conceived both naturally and through ART, it was unable to fully disentangle the contribution of ART to the observed PTB risk. Our subgroup analysis demonstrated that PCOS patients undergoing FET had a significantly higher risk of PTB, whereas those undergoing fresh embryo transfer did not exhibit a comparable increase. This discrepancy may be attributed to the use of high doses of estrogen and progesterone during endometrial preparation for FET, which could disrupt endometrial angiogenesis and immune regulation (59), thereby contributing to the elevated PTB risk. Further investigation is needed to clarify the mechanisms underlying these subgroup findings and to better understand the interplay between PCOS, ART, and PTB.

GDM, HDP, and gestational hypertension are complex, pregnancy-specific conditions involving multiple organ systems. Our meta-analysis revealed that women with PCOS had a substantially elevated risk of developing these complications, irrespective of whether conception occurred via FET or fresh embryo transfer. The underlying pathophysiology of PCOS is most commonly attributed to insulin resistance and hyperinsulinemia, with many women exhibiting insulin resistance independent of their BMI (60). During pregnancy, the inability to adequately compensate for this resistance leads to impaired glucose metabolism and intolerance (61). In the context of pregnancy, the additive effects of placental hormones exacerbate pre-existing insulin resistance (62), resulting in hyperglycemia and contributing to the increased prevalence of GDM in women with PCOS. Additionally, a recent meta-analysis encompassing both fresh and frozen embryo transfer cycles confirmed a heightened risk of pregnancy-induced hypertension among women with PCOS (51). Beyond the syndrome itself, hyperandrogenism may play an independent role in the development of hypertensive disorders (63). Elevated androgen levels, a hallmark of PCOS, have been implicated in vascular remodeling, including thickening of the carotid intima-media, which predisposes to hypertension (64). Other contributing factors warrant further exploration, including dyslipidemia, particularly elevated low-density lipoprotein (LDL) cholesterol levels, and persistent hyperinsulinemia. These factors may activate pro-inflammatory pathways, impair endothelial function, diminish vascular reactivity, and promote subclinical atherosclerosis (65).

Our analysis identified an elevated risk of PPROM among PCOS patients undergoing ART, while the likelihood of cesarean delivery was notably reduced. Comparable results were observed in the subgroup of patients who underwent FET. However, due to the limited number of available studies, we were unable to perform a subgroup analysis for those undergoing fresh embryo transfer. Further research is needed to further validate and support these findings. In addition, our meta-analysis found no significant differences in the risks of LBW, VLBW, macrosomia, SGA, VSGA, or LGA between PCOS and non-PCOS patients following ART. These results suggest that maternal PCOS may not exert a substantial influence on fetal or neonatal weight outcomes. Similarly, no significant difference in the risk of fetal malformation was observed between the two groups. Notably, as the majority of included studies focused on pregnancies achieved through FET, a clearer understanding of the associations between PCOS and fetal or neonatal weight, as well as fetal malformation, in fresh embryo transfer populations remains an important area for future investigation.

Our meta-analysis included only cohort studies, excluding case–control and cross-sectional studies, thereby providing robust evidence to clarify the causal association between PCOS and adverse pregnancy and perinatal outcomes. Furthermore, we prioritized the extraction of RRs and 95% CIs that had been adjusted for confounding factors in the included studies, which helped to minimize the influence of potential confounders on the final results. Additionally, we conducted subgroup analyses for each outcome based on the mode of ART, further exploring the impact of FET and fresh embryo transfer on the outcomes. However, this study has several limitations. First, the majority of the studies included in the final analysis were conducted in China, which limited the feasibility of performing subgroup analysis based on ethnicity and constrained the generalizability of our findings to the broader population. Second, only a limited number of studies have investigated the association between PCOS and adverse pregnancy and perinatal outcomes, such as miscarriage, HDP, and live birth rate, in patients undergoing fresh embryo transfer. This paucity of evidence has constrained our ability to perform subgroup analyses for fresh embryo transfer. Consequently, further research is urgently needed to better understand the differences in the associations between PCOS and adverse outcomes in the context of FET versus fresh embryo transfer. Third, the included studies varied in their adjustment for potential confounders, with some studies providing multivariable analyses that adjusted for factors such as maternal age and BMI, while others lacked adjustments for critical variables. This variability in study-level covariates may have contributed to the heterogeneity observed in certain outcomes and underscores the need for caution when interpreting the pooled estimates. Additionally, residual confounding remains a concern, as not all included studies adjusted for all critical covariates. These unmeasured confounders may partially explain the observed associations. Nevertheless, heterogeneity and sensitivity analyses indicated that most findings in our study were robust, with low heterogeneity and consistent reliability.

## 5 Conclusion

In conclusion, this study suggested that women with PCOS undergoing ART may have a higher clinical pregnancy rate and live birth rate compared with women without PCOS. However, these patients also appear to face notably increased risks of miscarriage, GDM, HDP, gestational hypertension, PPROM, PTB, and VPTB. Conversely, the risk of cesarean delivery might be lower in the PCOS group. No significant differences were observed between the PCOS and control groups regarding the risks of LBW, VLBW, macrosomia, SGA, VSGA, LGA, or fetal malformation. Similar findings were observed among patients undergoing FET. Further investigation is required to delineate the differential impact of PCOS on adverse outcomes in the context of FET versus fresh embryo transfer.

## Data Availability

The original contributions presented in the study are included in the article/Supplementary material, further inquiries can be directed to the corresponding author.
