# Supplementary material for: Adverse pregnancy and perinatal outcomes in women with polycystic ovary syndrome undergoing assisted reproductive technology: a systematic review and meta-analysis
Source: Front Med (Lausanne). 2025 Oct 10;12:1656389. doi: 10.3389/fmed.2025.1656389 (PMC12549646; doi:10.3389/fmed.2025.1656389)
Supplement: Supplementary file 1 [file Data_Sheet_1.DOCX]

| **PubMed 2534** |
| --- |
| #1 (polycystic ovary syndrome) OR (polycystic ovarian syndrome) OR (sclerocystic ovarian degeneration) OR (sclerocystic ovary syndrome) OR (Stein-Leventhal syndrome) OR (Stein Leventhal syndrome) OR (sclerocystic ovaries) OR (sclerocystic ovary) |
| #2 (pregnancy outcomes) OR (pregnancy outcome) OR (obstetric outcomes) OR (obstetric outcome) OR (reproductive outcomes) OR (reproductive outcome) OR (fertility outcomes) OR (fertility outcome) OR (placenta praevia) OR (placenta previa) OR (postpartum hemorrhage) OR (spontaneous hemoperitoneum) OR (antepartum hemorrhage) OR (pre-eclampsia) OR (preeclampsia) OR (gestational hypertension) OR (hypertensive disorders in pregnancy) OR (maternal hypertension) OR (pregnancy induced hypertension) OR (miscarriage) OR (stillbirth) OR (abruptio placentae) OR (placental abruption) OR (growth restriction) OR (gestational age) OR (livebirth rate) OR (preterm) OR (premature labor) OR (premature delivery) OR (premature birth) OR (preterm labor) OR (preterm delivery) OR (preterm birth) OR (caesarean) OR (cesarean section) OR (c-section) OR (small for gestational age) OR (low birth weight) OR (fertilisation rate) OR (fertilization rate) OR (clinical pregnancy rate) OR (intestinal perforation) OR (bowel perforation) OR (cholestasis) OR (mid-trimester loss) OR (implantation rate) OR (ovarian response) OR (cycle cancellation) |
| #3 (cohort studies) OR (cohort study) OR (prospective) OR (follow-up) OR (longitudinal) OR (retrospective) |
| #4 #1 AND #2 AND #3 |
| **Web of Science 3251** |
| #1 TS=((polycystic ovary syndrome) OR (polycystic ovarian syndrome) OR (sclerocystic ovarian degeneration) OR (sclerocystic ovary syndrome) OR (Stein-Leventhal syndrome) OR (Stein Leventhal syndrome) OR (sclerocystic ovaries) OR (sclerocystic ovary)) |
| #2 TS=((pregnancy outcomes) OR (pregnancy outcome) OR (obstetric outcomes) OR (obstetric outcome) OR (reproductive outcomes) OR (reproductive outcome) OR (fertility outcomes) OR (fertility outcome) OR (placenta praevia) OR (placenta previa) OR (postpartum hemorrhage) OR (spontaneous hemoperitoneum) OR (antepartum hemorrhage) OR (pre-eclampsia) OR (preeclampsia) OR (gestational hypertension) OR (hypertensive disorders in pregnancy) OR (maternal hypertension) OR (pregnancy induced hypertension) OR (miscarriage) OR (stillbirth) OR (abruptio placentae) OR (placental abruption) OR (growth restriction) OR (gestational age) OR (livebirth rate) OR (preterm) OR (premature labor) OR (premature delivery) OR (premature birth) OR (preterm labor) OR (preterm delivery) OR (preterm birth) OR (caesarean) OR (cesarean section) OR (c-section) OR (small for gestational age) OR (low birth weight) OR (fertilisation rate) OR (fertilization rate) OR (clinical pregnancy rate) OR (intestinal perforation) OR (bowel perforation) OR (cholestasis) OR (mid-trimester loss) OR (implantation rate) OR (ovarian response) OR (cycle cancellation)) |
| #3 TS=((cohort study) OR (cohort studies) OR (retrospective) OR (longitudinal) OR (follow-up) OR (prospective)) |
| #4 #1 AND #2 AND #3 |
| **The Cochrane Library 821** |
| #1 All Text=((polycystic ovary syndrome) OR (polycystic ovarian syndrome) OR (sclerocystic ovarian degeneration) OR (sclerocystic ovary syndrome) OR (Stein-Leventhal syndrome) OR (Stein Leventhal syndrome) OR (sclerocystic ovaries) OR (sclerocystic ovary)) |
| #2 All Text=((pregnancy outcomes) OR (pregnancy outcome) OR (obstetric outcomes) OR (obstetric outcome) OR (reproductive outcomes) OR (reproductive outcome) OR (fertility outcomes) OR (fertility outcome) OR (placenta praevia) OR (placenta previa) OR (postpartum hemorrhage) OR (spontaneous hemoperitoneum) OR (antepartum hemorrhage) OR (pre-eclampsia) OR (preeclampsia) OR (gestational hypertension) OR (hypertensive disorders in pregnancy) OR (maternal hypertension) OR (pregnancy induced hypertension) OR (miscarriage) OR (stillbirth) OR (abruptio placentae) OR (placental abruption) OR (growth restriction) OR (gestational age) OR (livebirth rate) OR (preterm) OR (premature labor) OR (premature delivery) OR (premature birth) OR (preterm labor) OR (preterm delivery) OR (preterm birth) OR (caesarean) OR (cesarean section) OR (c-section) OR (small for gestational age) OR (low birth weight) OR (fertilisation rate) OR (fertilization rate) OR (clinical pregnancy rate) OR (intestinal perforation) OR (bowel perforation) OR (cholestasis) OR (mid-trimester loss) OR (implantation rate) OR (ovarian response) OR (cycle cancellation)) |
| #3 All Text=((cohort studies) OR (cohort study) OR (prospective) OR (follow-up) OR (longitudinal) OR (retrospective)) |
| #4 #1 AND #2 AND #3 |
| **Embase 2745** |
| #1 'polycystic ovary syndrome'/exp OR 'polycystic ovarian syndrome' OR 'sclerocystic ovarian degeneration' OR 'sclerocystic ovary syndrome' OR 'stein-leventhal syndrome'/exp OR 'stein leventhal syndrome'/exp OR 'sclerocystic ovaries' OR 'sclerocystic ovary'/exp |
| #2 'pregnancy outcomes'/exp OR 'pregnancy outcome'/exp OR 'obstetric outcomes' OR 'obstetric outcome'/exp OR 'reproductive outcomes' OR 'reproductive outcome'/exp OR 'fertility outcomes' OR 'fertility outcome' OR 'placenta praevia'/exp OR 'placenta previa'/exp OR 'postpartum hemorrhage'/exp OR 'spontaneous hemoperitoneum'/exp OR 'antepartum hemorrhage'/exp OR 'pre eclampsia'/exp OR 'preeclampsia'/exp OR 'gestational hypertension'/exp OR 'hypertensive disorders in pregnancy' OR 'maternal hypertension'/exp OR 'pregnancy induced hypertension'/exp OR 'miscarriage'/exp OR 'stillbirth'/exp OR 'abruptio placentae'/exp OR 'placental abruption'/exp OR 'growth restriction' OR 'gestational age'/exp OR 'livebirth rate' OR preterm OR 'premature labor'/exp OR 'premature delivery'/exp OR 'premature birth'/exp OR 'preterm labor'/exp OR 'preterm delivery'/exp OR 'preterm birth'/exp OR caesarean OR 'cesarean section'/exp OR 'c section' OR 'small for gestational age'/exp OR 'low birth weight'/exp OR 'fertilisation rate' OR 'fertilization rate'/exp OR 'clinical pregnancy rate'/exp OR 'intestinal perforation'/exp OR 'bowel perforation'/exp OR 'cholestasis'/exp OR 'mid-trimester loss' OR 'implantation rate'/exp OR 'ovarian response'/exp OR 'cycle cancellation' |
| #3 'cohort studies'/exp OR 'cohort study'/exp OR prospective OR 'follow up'/exp OR 'longitudinal'/exp OR retrospective |
| #4 #1 AND #2 AND #3 |
