# Supplementary material for: Adverse pregnancy and perinatal outcomes in women with polycystic ovary syndrome undergoing assisted reproductive technology: a systematic review and meta-analysis
Source: Front Med (Lausanne). 2025 Oct 10;12:1656389. doi: 10.3389/fmed.2025.1656389 (PMC12549646; doi:10.3389/fmed.2025.1656389)

**FIGURE S1** Subgroup analysis of clinical pregnancy rate (A. Subgroup=Fresh/Frozen ET; B. Subgroup=Frozen ET) and miscarriage (C. Subgroup=Fresh ET; D. Subgroup=Frozen ET).


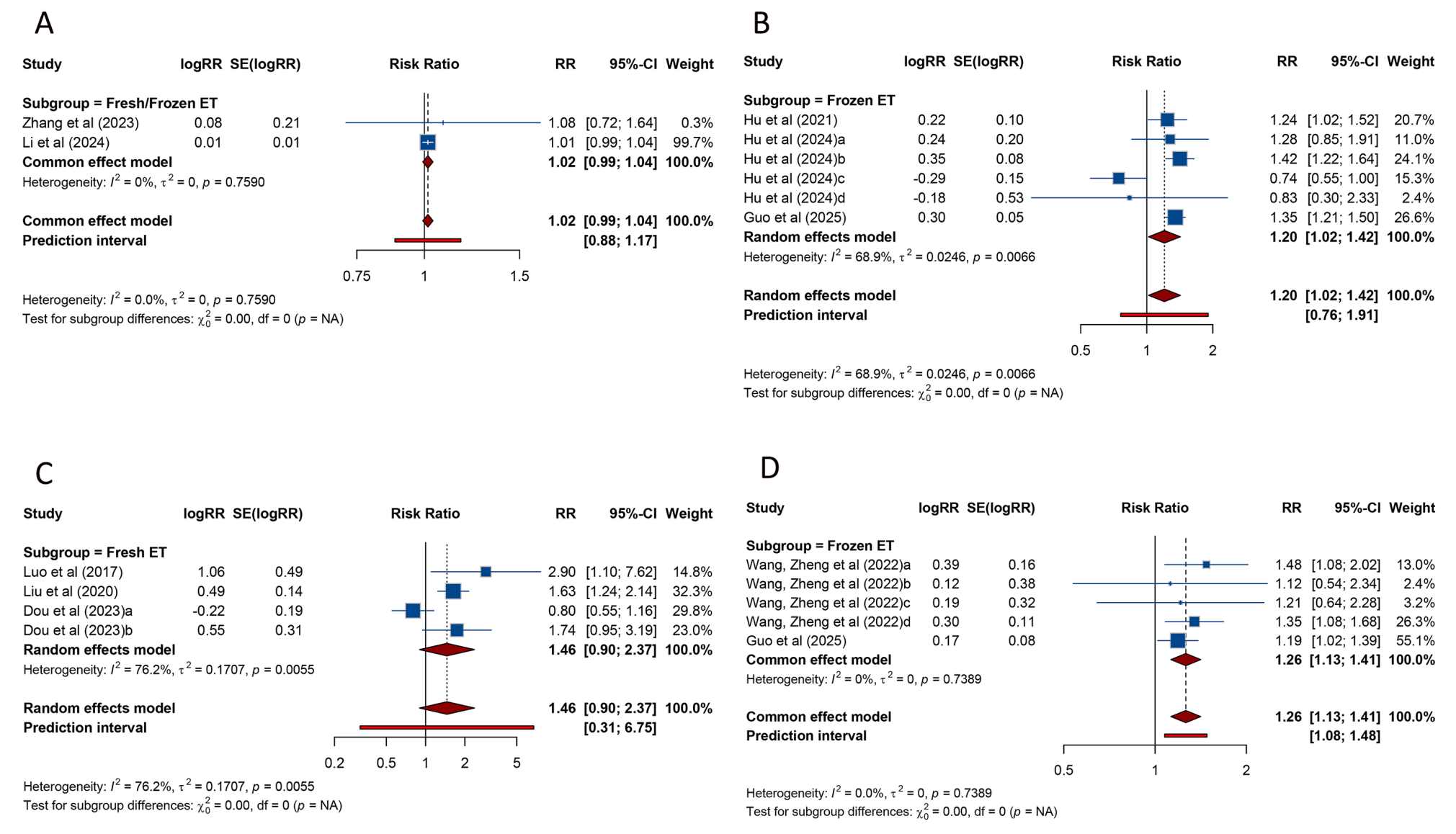


**FIGURE S2** Subgroup analysis of gestational diabetes mellitus (A. Subgroup=Frozen ET), hypertensive disorders of pregnancy (B. Subgroup=Fresh ET; C. Subgroup=Frozen ET), and gestational hypertension (D. Subgroup=Frozen ET).


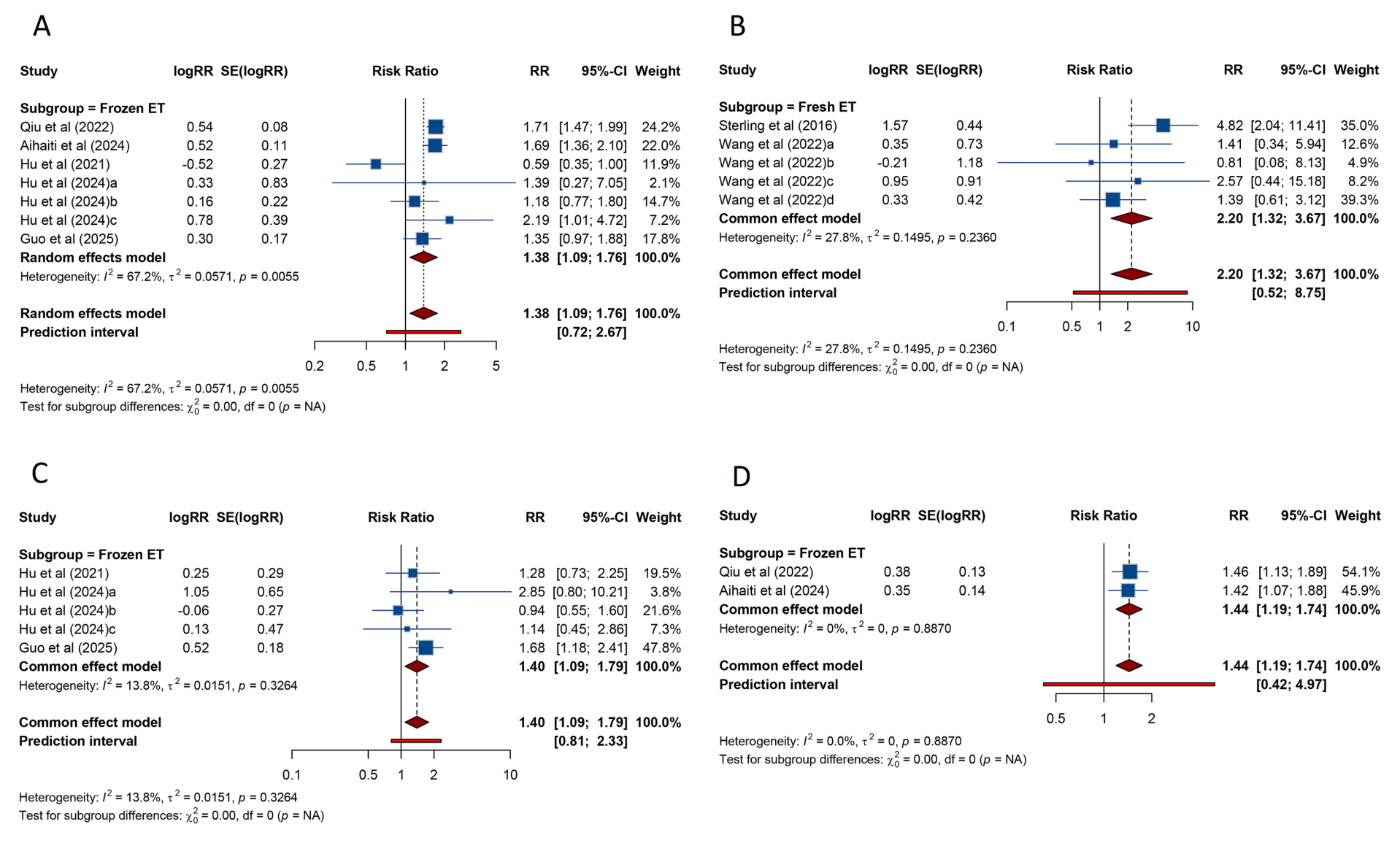


**FIGURE S3** Subgroup analysis of preterm premature rupture of membranes (A. Subgroup=Frozen ET) and caesarean delivery (B. Subgroup=Frozen ET).


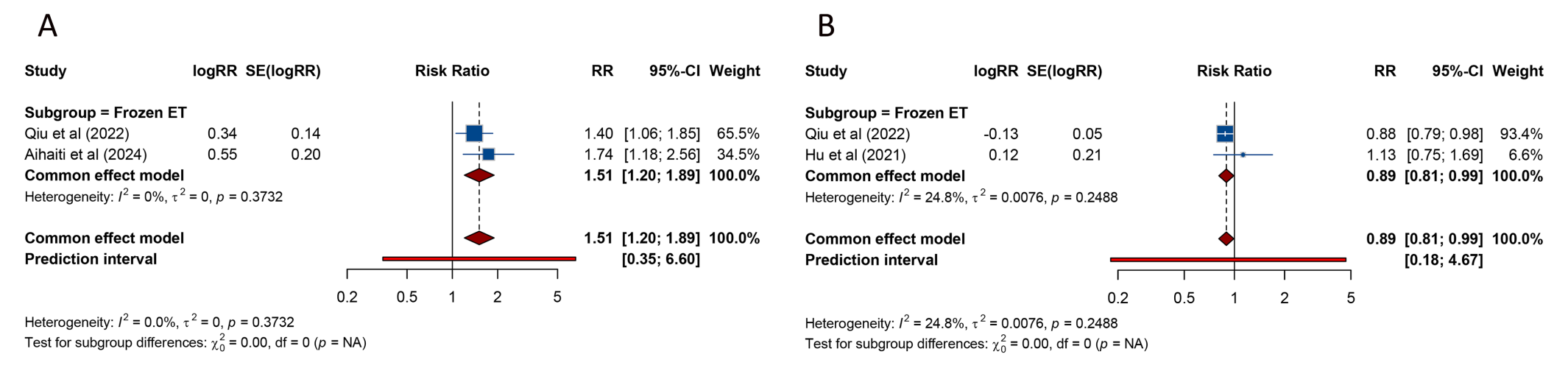


**FIGURE S4** Subgroup analysis of live birth rate. (A) Subgroup=Fresh/Frozen ET; (B) Subgroup=Fresh ET; (C) Subgroup=Frozen ET.


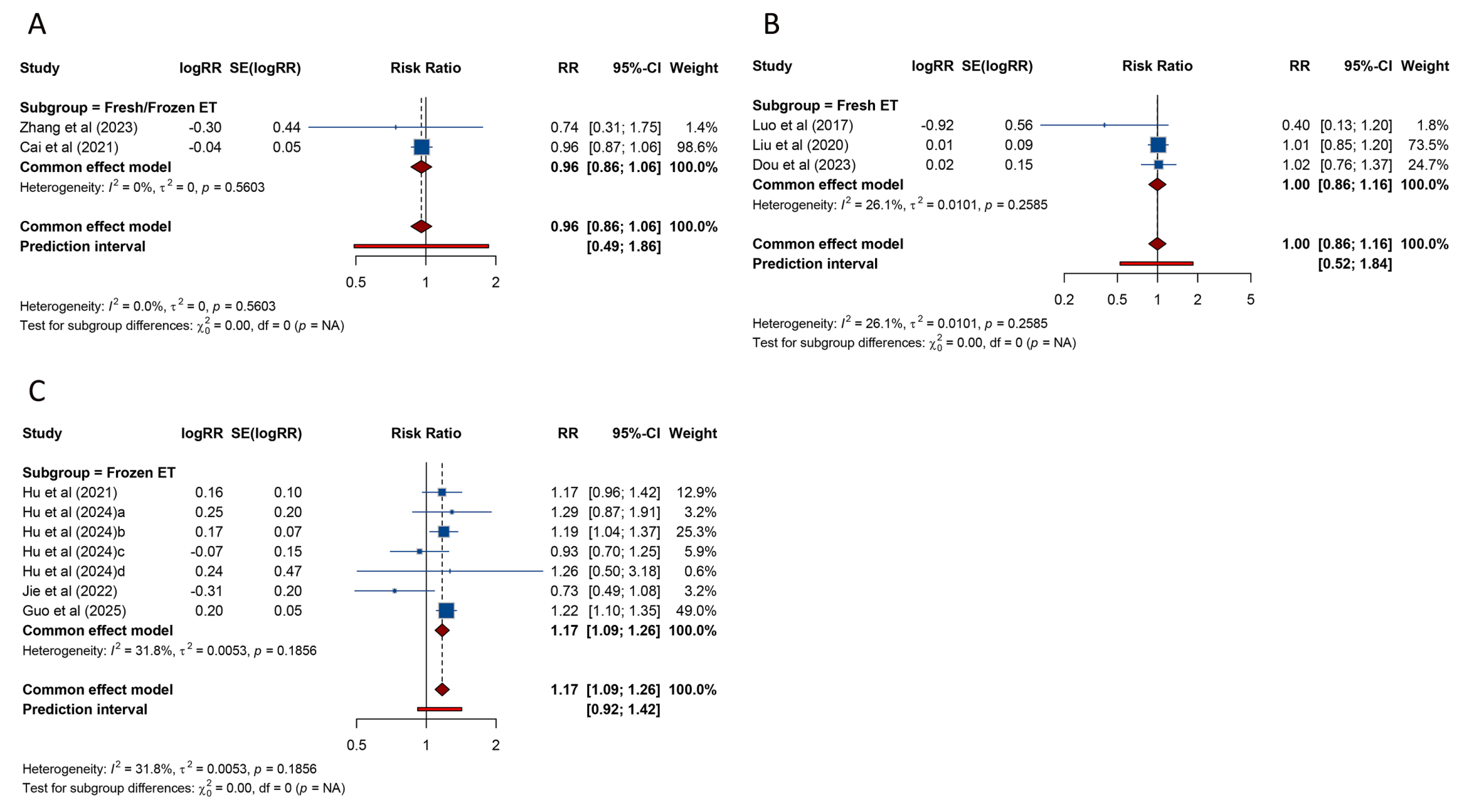


**FIGURE S5** Subgroup analysis of preterm birth (A. Subgroup=Fresh ET; B. Subgroup=Frozen ET) and very preterm birth (C. Subgroup=Fresh ET; D. Subgroup=Frozen ET).


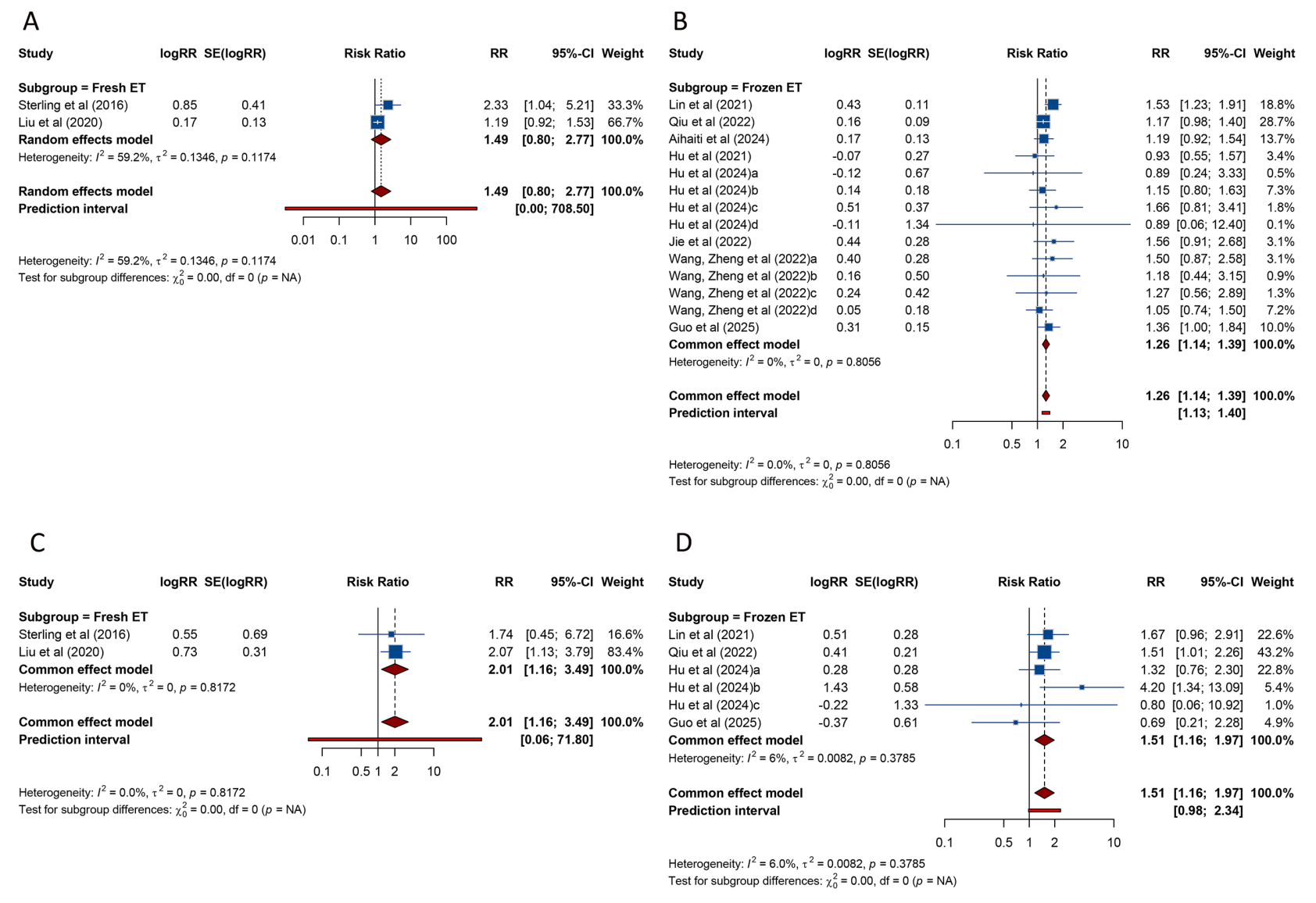


**FIGURE S6** Subgroup analysis of low birth weight (A. Subgroup=Frozen ET), very low birth weight (B. Subgroup=Frozen ET) and macrosomia (C. Subgroup=Frozen ET).


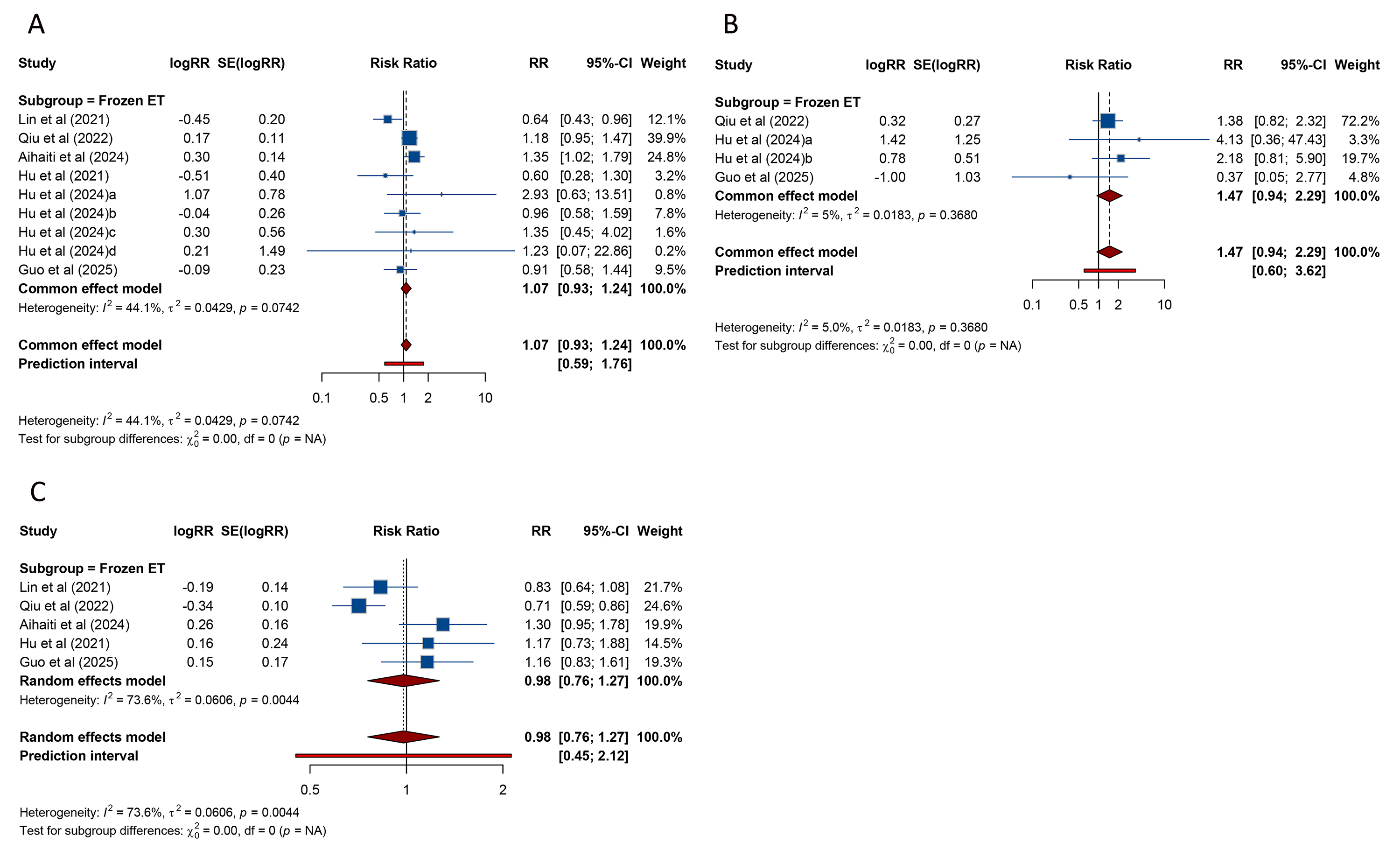


**FIGURE S7** Subgroup analysis of small for gestational age (A. Subgroup=Frozen ET), very small for gestational age (B. Subgroup=Frozen ET), large for gestational age (C. Subgroup=Frozen ET) and fetal malformation (D. Subgroup=Frozen ET).


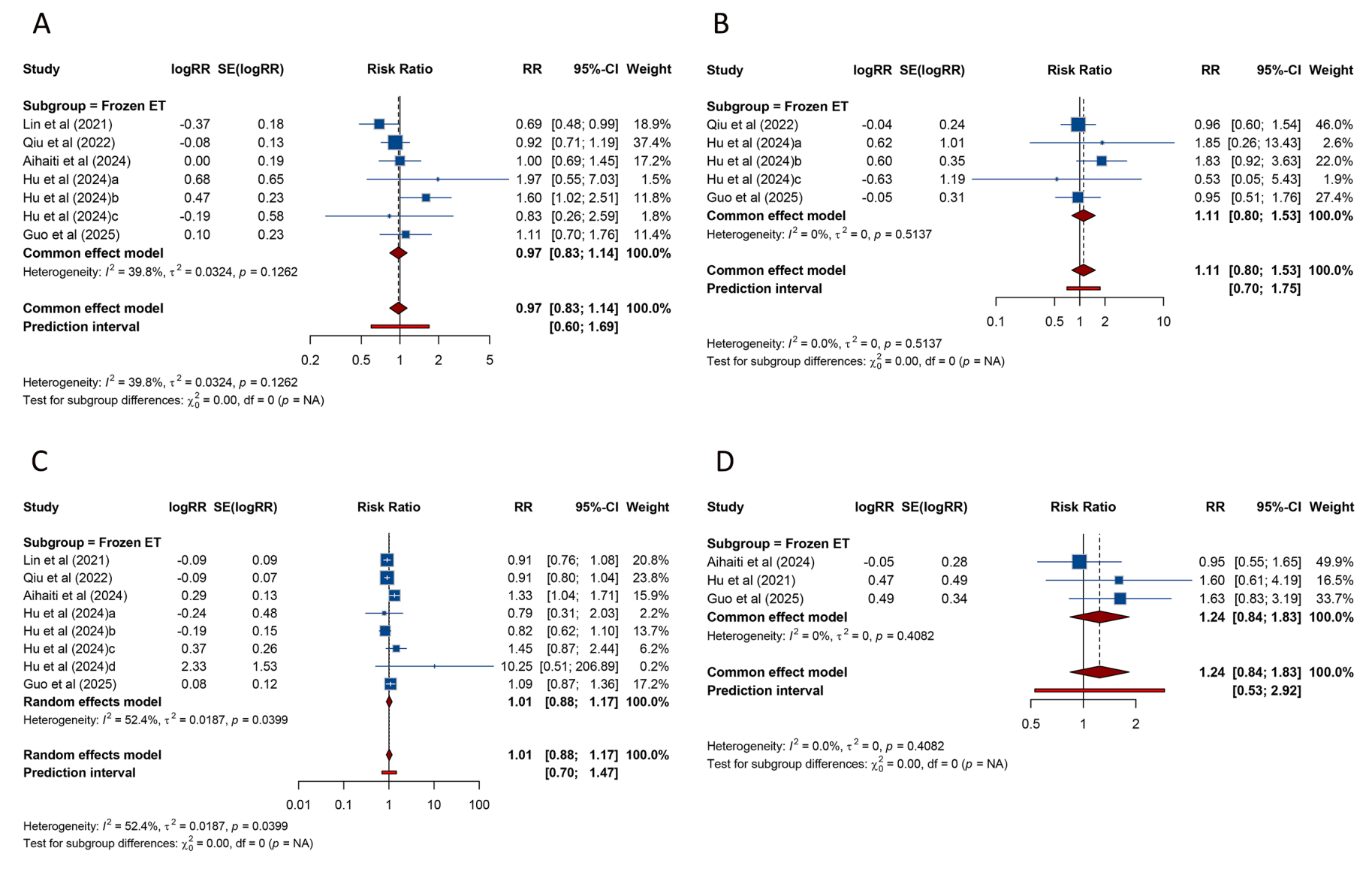


**FIGURE S8** Sensitivity analysis for the pooled results of clinical pregnancy rate (A), miscarriage (B), hypertensive disorders of pregnancy (C), live birth rate (D), preterm birth (E), and low birth weight (F).


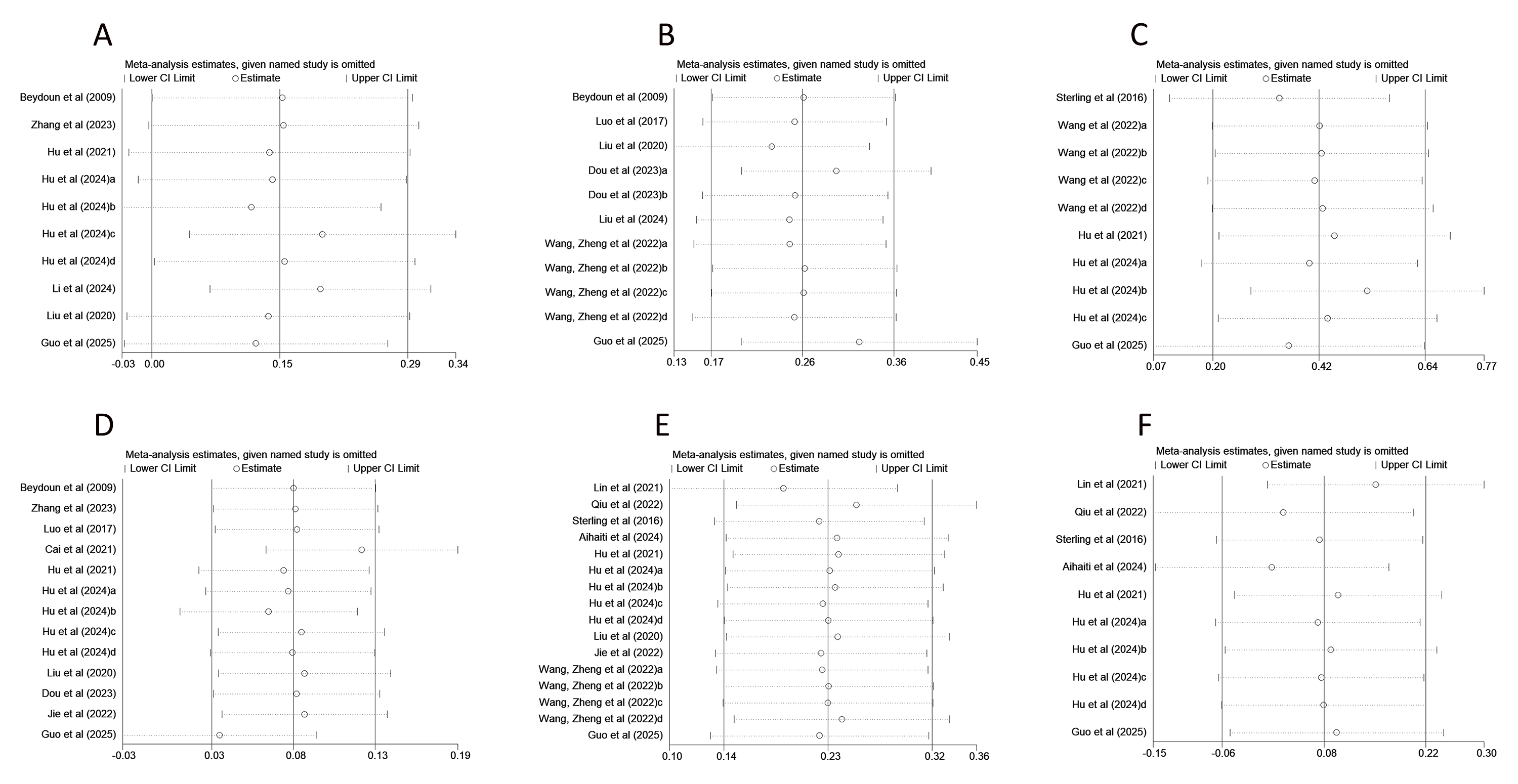


**FIGURE S9** Funnel plots of the pooled results of clinical pregnancy rate (A), miscarriage (B), hypertensive disorders of pregnancy (C), live birth rate (D), preterm birth (E), and low birth weight (F).


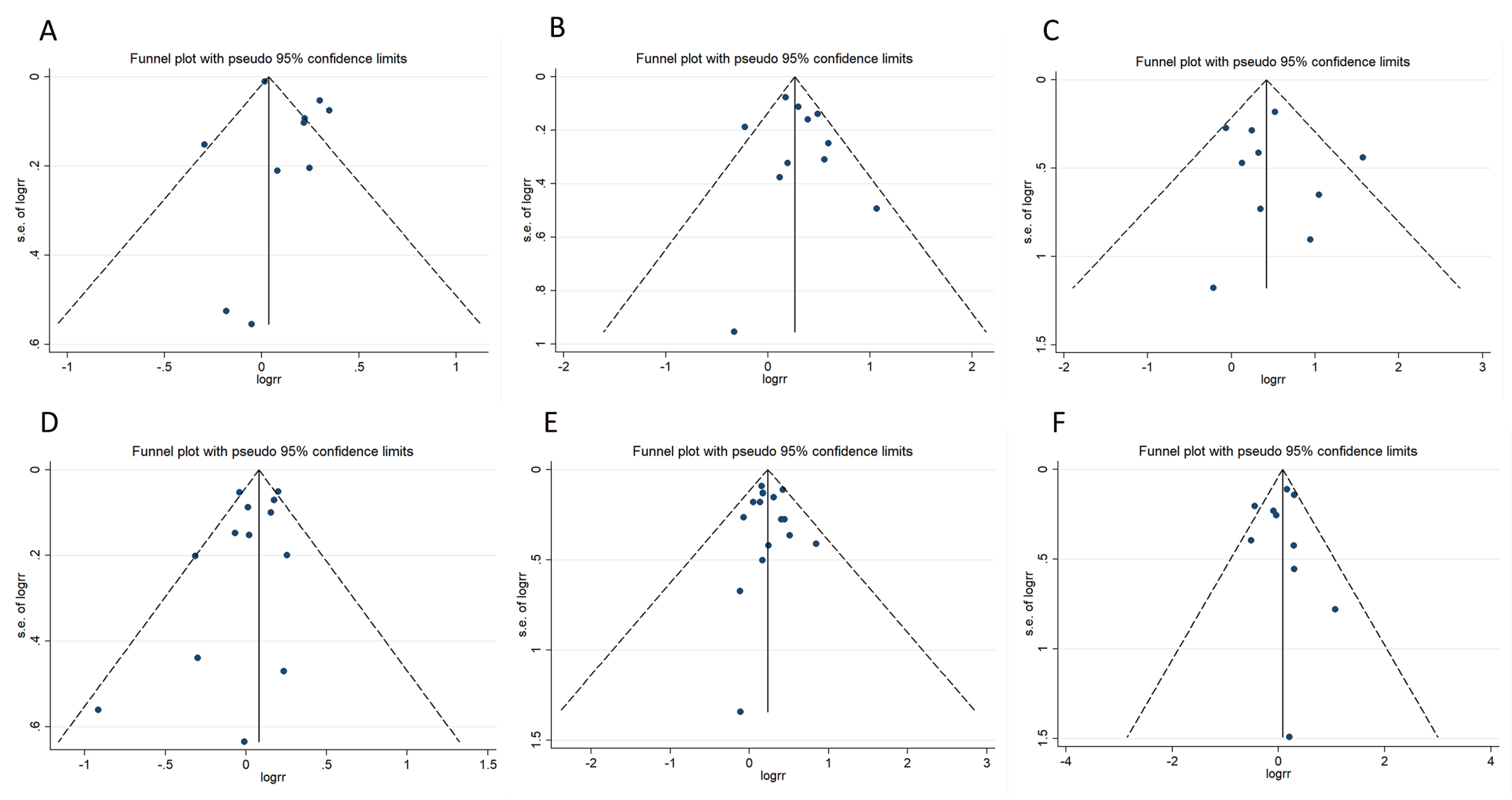

Supplement: Supplementary file 2 [file Data_Sheet_2.DOCX]
