# Supplementary material for: Adverse pregnancy and perinatal outcomes in women with polycystic ovary syndrome undergoing assisted reproductive technology: a systematic review and meta-analysis
Source: Front Med (Lausanne). 2025 Oct 10;12:1656389. doi: 10.3389/fmed.2025.1656389 (PMC12549646; doi:10.3389/fmed.2025.1656389)
Supplement: Supplementary file 3 [file Table_1.DOCX]

| TABLE S1 The Newcastle-Ottawa quality assessment scale of the included cohort studies. | | | | | | | | | | | | |
| --- | --- | --- | --- | --- | --- | --- | --- | --- | --- | --- | --- | --- |
| Study | Selection | | | |  | Comparability | |  | Assessment of outcome | | | Total score |
|  | Representativeness of exposure arm(s) | Selection of the comparative arm(s) | Origin of exposure source | Demonstration that outcome of interest was not present at start of study |  | Studies controlling the most important factors | Studies controlling the other main factors |  | Assessment of outcome with independency | Adequacy of follow-up length | Lost to follow-up acceptable |  |
| Lin et al (2021) | 1 | 1 | 1 | 1 |  | 1 | 0 |  | 1 | 1 | 1 | 8 |
| Qiu et al (2022) | 1 | 1 | 1 | 1 |  | 1 | 0 |  | 1 | 1 | 1 | 8 |
| Sterling et al (2016) | 1 | 1 | 1 | 1 |  | 1 | 0 |  | 1 | 1 | 1 | 8 |
| Aihaiti et al (2024) | 1 | 1 | 1 | 1 |  | 1 | 1 |  | 1 | 1 | 1 | 9 |
| Beydoun et al (2009) | 0 | 1 | 1 | 1 |  | 1 | 0 |  | 1 | 1 | 1 | 7 |
| Zhang et al (2023) | 1 | 1 | 0 | 1 |  | 1 | 0 |  | 1 | 1 | 1 | 7 |
| Wang et al (2022) | 1 | 1 | 1 | 1 |  | 1 | 0 |  | 1 | 1 | 1 | 8 |
| Luo et al (2017) | 0 | 1 | 1 | 1 |  | 1 | 1 |  | 1 | 1 | 1 | 8 |
| Cai et al (2021) | 1 | 1 | 1 | 1 |  | 1 | 0 |  | 1 | 1 | 1 | 8 |
| Hu et al (2021) | 1 | 1 | 1 | 1 |  | 1 | 1 |  | 1 | 1 | 1 | 9 |
| Hu et al (2024) | 1 | 1 | 1 | 1 |  | 0 | 0 |  | 1 | 1 | 1 | 7 |
| Li et al (2024) | 1 | 1 | 1 | 1 |  | 1 | 0 |  | 1 | 1 | 1 | 8 |
| Liu et al (2020) | 1 | 1 | 1 | 1 |  | 1 | 0 |  | 1 | 1 | 1 | 8 |
| Dou et al (2023) | 1 | 1 | 1 | 1 |  | 1 | 1 |  | 1 | 1 | 1 | 9 |
| Liu et al (2024) | 1 | 1 | 1 | 1 |  | 1 | 0 |  | 1 | 1 | 1 | 8 |
| Jie et al (2022) | 1 | 1 | 1 | 1 |  | 1 | 1 |  | 1 | 1 | 1 | 9 |
| Wang, Zheng et al (2022) | 1 | 1 | 1 | 1 |  | 1 | 0 |  | 1 | 1 | 1 | 8 |
| Guo et al (2025) | 1 | 1 | 1 | 1 |  | 1 | 0 |  | 1 | 1 | 1 | 8 |
